# The adoption of international travel measures during the first year of the COVID-19 pandemic: a descriptive analysis

**DOI:** 10.1186/s12992-024-01071-7

**Published:** 2024-10-04

**Authors:** Karen A. Grépin, Mingqi Song, Julianne Piper, Catherine Z. Worsnop, Kelley Lee

**Affiliations:** 1 School of Public Health, Li Ka Shing Faculty of Medicine, University of Hong Kong, Pokfulam, Hong Kong SAR China; 2 Independent, Xuhui District, 200030 China; 3 Simon Fraser University, Vancouver, BC V5N 3V2 Canada; 4 School of Public Policy, University of Maryland, College Park, MD 20742 USA

## Abstract

**Objective:**

To describe the adoption of international travel measures during the first year of the COVID-19 pandemic.

**Methods:**

To comprehensively analyze the measures adopted, we constructed a dataset based on the WHO’s Public Health and Social Measures (PHSM) database, which covered 252 countries, territories, or other areas (CTAs), including all 194 WHO Member States, from December 31, 2019, to December 31, 2020. We examined the adoption of measures by type, over time, and by the implementing and targeted CTA, including their levels of income.

**Findings:**

We identified 11,431 international travel measures implemented during the first year of the pandemic. The adoption of measures was rapid and widespread: over 60% of Member States had adopted a travel measure before the WHO declared COVID-19 a Public Health Emergency of International Concern on January 30, 2020. Initially, health screening and travel restrictions were the most adopted measures; however, quarantine and testing became more widely adopted over time. Although only a small portion of the total measures adopted constituted full border closure, approximately half of all Member States implemented this measure. Many travel measures targeted all CTAs but were unlikely to have been adopted universally enough to provide public health benefits. Low-income countries relied more on more universal measures, including full border closure, and were slower in scaling up testing compared to higher-income countries.

**Conclusion:**

The adoption of international travel measures during the first year of the COVID-19 pandemic varied across jurisdictions and over time. Lower-income countries used a different mix and scaled-up measures slower than higher-income countries. Understanding what measures were used is crucial for assessing their effectiveness in controlling the spread of COVID-19, reviewing the usefulness of the International Health Regulations, and informing future pandemic preparedness and response activities.

**Supplementary Information:**

The online version contains supplementary material available at 10.1186/s12992-024-01071-7.

## Introduction

The COVID-19 pandemic has had profound health, economic, and social consequences globally. Faced with enormous uncertainty at the start of the pandemic, as well as the lack of an effective vaccine or treatment, most governments adopted measures aimed at mitigating the effects of the virus on their population. Of the measures adopted, the use of international travel measures was among the most controversial. Not only did the implementation of such measures contribute to major reductions in international traffic and trade, leading to severe disruptions to societies and economies, but the use of such measures intersected with concepts of sovereignty, security, and global governance. The effectiveness, equity, legality, and ethics of the adoption of these measures during the pandemic were questioned and continue to be debated [1, 2].

At the onset of the pandemic, it was widely believed that international travel measures, in particular travel restrictions and border closures, were ineffective as public health measures and their adoption was in violation of international law [3, 4]. When the World Health Organization (WHO), upon the advice of the Emergency Committee, declared COVID-19 a public health emergency of international concern (PHEIC) in late January 2020, it did not recommend the adoption of travel or trade restrictions, citing limited scientific evidence of their effectiveness [5]. This did not, however, stop the rapid and widespread adoption of such restrictions and other travel measures in early 2020. Indeed, there is evidence that the PHEIC declaration, as well as the announcement made by the WHO a few weeks later that COVID-19 met the criteria of a pandemic, prompted many countries to adopt such measures [6]. While our understanding of the effectiveness of international travel measures has evolved [7–9], with evidence now supporting the view that some types of measures, especially quarantine in combination with testing, when applied early and universally enough, might warrant their use to achieve public health goals, the broader economic and social consequences of these measures remain poorly understood [10–12]. As such, whether, what, when and how a country should adopt international travel measures during future public health emergencies remains unclear. The rationale to inform decision making on such measures will also differ across countries given different public health objectives (e.g., elimination vs. mitigation), tolerance for economic and social disruptions, normative frameworks (e.g., priority given to health and social equity), and capacity to implement.

The use of international travel measures during outbreaks of infectious disease with epidemic or pandemic potential is far from new. While the International Health Regulations (IHR2005) aim to limit the unnecessary use of international travel (and trade) measures—in part because these measures can disincentivize rapid and transparent outbreak reporting [13], the proportion of countries adopting such measures during the COVID-19 pandemic was much higher than for other major outbreaks since the IHRs were revised in 2005. For example, during the H1N1 influenza pandemic (2009) and the outbreak of Ebola Virus Disease in West Africa (2014-16), 25–33% of countries adopted international travel measures [14, 15].

During COVID-19, there was also a more diverse range of international travel measures adopted than in the past, including advisories and warnings, symptomatic screening, restrictions based on the source location or other traveler characteristics, border closures, diagnostic testing, quarantine measures, and immunity certification [16]. During previous major outbreaks, countries largely adopted advisories and warnings, screening, and targeted restrictions. As a prolonged pandemic, more countries were targeted, measures were implemented over a longer period, and governments frequently modified their international travel measures. The use of specific types of measures adopted by countries has thus far not been well described, due in part to a lack of a standardized typology of measures and data to quantify and track their adoption.

Understanding which types of international travel measures were adopted, when, and how by which jurisdictions during the COVID-19 pandemic is important for many reasons. It could help us to better understand the effectiveness of travel measures. Existing reviews of the literature on this topic have recognized the challenges of attribution due to an incomplete view of the specific and full range of travel measures that were in place at any given time [9]. It can also improve the relevancy of the IHRs and inform ongoing negotiations of a potential Pandemic Agreement. Moreover, given the likelihood that such measures will be used again during future infectious disease outbreaks, a quantitative analysis of travel measures could inform future pandemic planning and response activities.

A small number of studies have attempted to track and monitor the adoption of international travel measures during the pandemic. For example, the COVID border accountability project (COBAP), developed a database of border closure measures [17]. Using their dataset, the authors found a large increase in the number of countries adopting full or partial border closures in mid-March 2020. Piccoli et al. (2021) developed the Citizenship, Migration, and Mobility in a Pandemic (CMMP) dataset, which tracked a subset of the measures captured in our dataset starting in early March 2020 [18]. This dataset also showed a large increase in the adoption of measures in mid-March and that the application of measures became more universal over time. Finally, the International Organization on Migration (IOM), in collaboration with the International Air Transport Association (IATA), assembled the Global Mobility Restrictions Overview, which tracked the status of a set of travel measures implemented from March 2020 to January 2023. While these datasets and studies allow us to better understand the adoption of international travel measures during the pandemic, all focus on a narrow set of measures (e.g. border closure) and/or limited time periods (e.g. after March 2020). They also do not systematically track the targets of the measures adopted. The lack of standardized and consistently applied terminology and typology of measures by these studies also limits comparisons across countries and datasets.

In this paper, we build upon the existing literature to construct a dataset of a fuller range of international travel measures adopted during the first year of the COVID-19 pandemic. We then investigate and describe major patterns and trends in the adoption of these measures by type, jurisdiction of adoption, target, and timing. Based on these findings, we discuss the implications of the adoption of international travel measures to the IHRs and to inform future pandemic preparedness and response.

## Methods

To describe the number, type, and targets of international travel measures adopted during the first year of the pandemic, we constructed a dataset based on previously generated COVID-19 policy response trackers [19]. We first identified and compared leading trackers to understand their coverage of travel measures (see Sect. 1 of the Appendix). The Public Health and Social Measures (PHSM) database, which was coordinated by WHO, was selected as our primary source of data because it consolidated many trackers into a single database, collected data at the individual travel measure-level beginning in December 2019, and was the most credible database available. Despite WHO efforts to harmonize variables and data across the underlying trackers, PHSM did not apply standardized terminology to the different types and subtypes of international travel measures captured in the underlying datasets. It also lacked data on the targeted jurisdiction(s) and its travel measure-level data had not been validated (e.g., implementation date and type of measure).

Based on a critical review of terminology [16], we defined an international travel measure used in the context of COVID-19 as an action taken to control the movement of people across two or more national jurisdictions with the stated intent of preventing, controlling, or mitigating travel-related public health risks. Using the April 20, 2021, version of PHSM, we captured measures implemented by 237 countries/territories/areas (CTAs) from the start of the pandemic in late December 2019 to the end of 2020 (roughly the first year of the pandemic). A CTA was defined as any officially assigned ISO-3166 country or country subdivision code, which includes countries, overseas territories, and other special administrative regions or territories with unique political or administrative arrangements, and the ability to adopt their own travel measures. Please see Appendix Fig. 1 and Table 3 for a more detailed description of our taxonomy and how we defined the different types of measures in our dataset. Our dataset initially included the 234 CTAs identified in the PHSM database, but we then split out Hong Kong, Taiwan, and Macao from Mainland China as they have their own ISO-3166 code and have independently implemented travel measures. There are 249 officially recognized CTAs in ISO-3166, however, we further split out the Dutch islands of Bonaire, Sint Eustatius, and Saba as they were distinct in PHSM and had not implemented the same measures, for a total of 252 potential CTA . We trimmed the dataset to measures implemented from December 31, 2019, to December 31, 2020, which we refer to as the first year of the pandemic for simplicity. We focused on this period because this was when there was the most intense use of international travel measures. Plus, tracking and reporting of such measures was generally reliable during the first year. We became less confident in the quantity and quality of reporting of measures over time, likely due to reporting fatigue, and limited capacity to track the sheer volume and frequent adaptation of measures. Some of the underlying datasets that comprise the PHSM also ceased collecting data around this period [19].

A protocol was developed to code and validate the PHSM data using consistent terminology. A team of coders was trained on the protocol and each coder was assigned a set of CTAs, for which they would code all measures in PHSM. New measures were added to our dataset under one of two scenarios. First, while reading websites linked as sources to the PHSM database, coders sometimes identified previously unrecorded travel measures, which were then added. Second, if a measure in the PHSM database included more than one measure type according to our travel measure taxonomy (see Appendix Fig. 1), the original entry was split so that there was one entry in our dataset for each travel measure type. Compared to PHSM, new records accounted for 40% of our dataset.

Each entry in our dataset records the implementing CTA, measure type and subtype, one or more targeted CTA(s), and the date of adoption. Measure type refers to the broad measure type in our taxonomy, such as testing or border closure. Measure subtype provides specific details such as the timing of testing (e.g., pre-arrival) or whether a CTA closed its land, sea, air, or all borders. Appendix Tables 3 and 4 provide a complete list of measure types and subtypes. In PHSM, the status changes of measures were reported, which included the introduction of a new measure, the strengthening or extension of existing measures, the lifting or finishing of measures, and other changes (see Appendix Table 5 for more details). While each of these changes were recorded as an individual measure in our dataset, our data quality analysis determined that it was not always possible to say with certainty that measures tagged as new were in fact new or that other changes were also valid. As such, we also present what we define as the “earliest” measure, or the first time we see a country introducing a particular measure type against a particular CTA target. A version of Fig. 3, which excludes measures “extending” and “finishing” is also included in the Appendix. It is also for this reason that we present cumulative incidence figures, because if we observed a particular type of measure in the dataset, it was very likely to have occurred but we have less confidence around changes to measures or the easing or ending of measures.

If the PHSM data on a measure was unclear, for example, if the original internet link was broken or if the details of a measure could not easily be obtained, then the coder would flag the measure for additional research and discussion. Flagged measures were examined by a second coder who followed an extended coding protocol, including searching online for alternative websites to clarify the details of the measure. If the coder still could not decide on a course of action, a decision was made by the lead coder on which data to keep or by a discussion with the first author of this paper.

To identify the targets of the measures, we imported our dataset into STATA18 and then extracted the targeted country, region, city, province, or other jurisdiction that had been identified in our coding process. Most measures targeted an entire CTA, which was mapped as the target for those measures. However, if a measure targeted only a sub-jurisdiction, for example, a city (e.g., Wuhan) or a province (e.g., Hubei), then we flagged these as sub-CTA measures and then mapped it to the CTA-level target (e.g., China). Many measures targeted all CTAs or inbound travelers, in which case we flagged this as a measure that targeted “all CTAs” and mapped it to all 252 potential CTAs except the implementing CTA as targets. Similarly, some measures targeted all CTAs except a shorter list of CTAs, in which case we mapped to all CTAs except the implementing and exempted CTAs as targets.

Occasionally, a measure described a specific regional organization as its target (e.g., the European Union or the Gulf Cooperation Council), in which case we used official lists as of 2020 to map these measures to member CTAs. However, if a measure targeted a vague region, for example, “Asian countries”, we did not map these measures to individual CTAs due to the potential to misinterpret the actual target. Approximately 8.4% of the measures in our database were not mapped to a CTA. Some measures targeted specific types of travelers (e.g., non-essential workers), in which case we mapped these targets to all CTAs (unless more information was given) but flagged these as individual-based measures. Full details of the mapping, including the reasons some measures remained unmapped, can be found in Appendix Table 9.

We further categorized the implementing and targeted CTAs into the 194 WHO Member States. The remaining CTAs, collectively referred to as non-Member State CTAs, were mostly island territories but also included Lichtenstein and semi-autonomous regions, such as Hong Kong, Taiwan, Kosovo, and Palestine. We also categorized the income level of Member States using the World Bank’s income classification scheme of 2022–2023. Two Member States, Niue and Cook Islands, lacked income data, but we defined them as upper-middle-income and high-income, respectively.

In our figures, measures were grouped by week of implementation where the start of the first week of 2020 is defined as Sunday, December 29, 2019. The PHEIC was declared in week 5 and WHO described COVID-19 as a pandemic in week 11. A detailed description of the full steps taken to construct our dataset is available in the Appendix. As the project relied exclusively on secondary data in the public domain, we did not seek ethical approval for this project.

## Results

Table 1 summarizes the number of individual measures reported as adopted during the first year of the pandemic. Column 1 describes all measures adopted in 2020, column 3 describes the earliest observed measure for each implementing CTA and measure type (e.g., the first time Australia adopted quarantine), and column 5 describes measures adopted during the first six months of 2020 (i.e., through June 30, 2020). The earliest measure in our database, a measure adopted by Taiwan targeting travelers from Wuhan, was recorded on December 31, 2019, the same day that China CDC notified the WHO of a cluster of pneumonia cases of unknown origin.

**Table 1:** Summary of measures

		(1)	(2)	(3)	(4)	(5)	(6)
		Full Year		Earliest		Half Year	
Category		All	% of Total	All	% of Total	All	% of Total
		*n*	%	*n*	%	*n*	%
Total number of measures		11,431	100	2,164	18.9	5,693	49.8
Implementing CTAs		237		237		237	
**Measure source**							
Measure in the original PHSM dataset		7,003	61.3	1,452	67.1	4,010	70.4
Newly added measure		4,428	38.7	712	32.9	1,683	29.6
**Total**		**11**,**431**	**100**	**2**,**164**	**100**	**5**,**693**	**100**
**Measure implementation date**							
Earliest date		31dec2019		31dec2019		31dec2019	
Mean date		01jul2020		06apr2020		04apr2020	
Latest date		31dec2020		31dec2020		30jun2020	
**Measure type**							
Quarantine		2,488	21.8	248	11.5	1,129	19.8
Testing		2,015	17.6	270	12.5	380	6.7
Individual-based restrictions		1,580	13.8	230	10.6	920	16.2
Restricting flights		1,270	11.1	221	10.2	722	12.7
Health screening		779	6.8	207	9.6	580	10.2
Additional travel documents		588	5.1	175	8.1	227	4.0
CTA-based restrictions		576	5.0	139	6.4	299	5.3
Travel advice or warning		527	4.6	138	6.4	365	6.4
Border closure		390	3.4	127	5.9	254	4.5
Other travel restrictions		352	3.1	152	7.0	263	4.6
Visa-related restrictions		295	2.6	94	4.3	190	3.3
Other types		571	5.0	163	7.5	364	6.4
**Total**		**11**,**431**	**100**	**2**,**164**	**100**	**5**,**693**	**100**

Notes: Columns 1 and 5 describe all measures while column 3 describes the first observed measure for each implementing country and measure type [i.e. the first time Australia adopted quarantine]. Columns 1 and 3 cover measures implemented from December 31, 2019, to December 31, 2020. Column 5 covers measures implemented from December 31, 2019, to June 30, 2020

In total, we observed 11,431 reported measures in 2020, of which 61.3% were in the original PHSM dataset. The remaining measures were identified by following our coding procedures described above. About half of the measures (5,693) were adopted in the first half of 2020. The mean date of adoption of measures in 2020 was July 1, while the mean date of adoption for the earliest measures was April 6.

In 2020, quarantine was the most adopted measure type (21.8%), followed by testing (17.6%), individual-based travel restrictions or bans (13.8%), and suspending or restricting flights (11.1%). Quarantine was also the most adopted measure type during the first half of 2020 (19.8%). Testing was less common (6.7%) during the first half, while individual-based travel restrictions or bans (16.2%), suspending or restricting flights (12.7%) and health screenings (10.2%) were more commonly adopted. Only a small percentage (3.5%) of the measures adopted in 2020 were defined as full border closure.

In Table 2, we describe the implementing and targeted CTAs. High-income Member States adopted 41.7% of the measures in 2020 but accounted for a lower proportion (36.6%) of implementing CTAs in the first half of the year. The trend was reversed for upper-middle-income and lower-middle-income Member States. Upper middle-income states adopted 24.1% of measures in 2020 and 26.2% of measures in the first half of the year while lower middle-income states adopted 17.2% of measures in 2020 but 19.3% in the first half of the year. Low-income states adopted about the same proportion of the measures in the first half and the full year. Non-WHO Member State CTAs adopted 8.9% of measures.

**Table 2:** Summary of measures by implementing and targeted CTAs

		(1)	(2)	(3)	(4)	(5)	(6)
		Full Year		Earliest		Half Year	
Category		All	% of Total	All	% of Total	All	% of Total
		*n*	%	*n*	%	*n*	%
Total measures		11,431	100	2,164	18.9	5,693	49.8
Implementing CTAs		237		237		237	
**Implementing CTA Income Level**							
High income (*n* = 61)		4,772	41.7	637	29.4	2,082	36.6
Upper middle income (*n* = 55)		2,754	24.1	550	25.4	1,491	26.2
Lower middle income (*n* = 49)		1,964	17.2	438	20.2	1,099	19.3
Low income (*n* = 29)		921	8.1	243	11.2	473	8.3
No income data (*n* = 40)		1,020	8.9	296	13.7	548	9.6
**Total**		**11**,**431**	**100.0**	**2**,**164**	**100**	**5**,**693**	**100**
**Implementing CTAs**							
EURO		4,451	38.9	584	27.0	2,067	36.3
WPRO		1,525	13.3	254	11.7	838	14.7
PAHO		1,332	11.7	310	14.3	648	11.4
AFRO		1,538	13.5	409	18.9	769	13.5
EMRO		995	8.7	203	9.4	486	8.5
SEARO		570	5.0	108	5.0	337	5.9
**Total Member States**		10,411	91.1	1,868	86.3	5,145	90.4
Non-Member State CTAs		1,020	8.9	296	13.7	548	9.6
**Total**		11,431	100	2,164	100	5,693	100
**Targeted CTAs**							
All CTAs		5,588	48.9	1,087	50.2	2,806	49.3
Member States		4,765	41.7	877	40.5	2,367	41.6
Only Non-Member State CTAs		81	0.7	13	0.6	38	0.7
**Total mapped**		10,434	91.3	1,977	91.4	5,211	91.5
Unmapped		997	8.7	187	8.6	482	8.5
**Total**		11,431	100	2,164	100	5,693	100
**Targeted Member States**							
EURO		3,268	28.6	399	18.4	1,458	25.6
WPRO		2,063	18.0	554	25.6	1,259	22.1
EMRO		1,407	12.3	244	11.3	771	13.5
PAHO		1,061	9.3	116	5.4	352	6.2
SEARO		840	7.3	88	4.1	293	5.1
AFRO		728	6.4	66	3.0	203	3.6
**Total**		9,367		1,467		4,336	

Notes: Columns 1 and 5 describe all measures while column 3 describes the first observed measure for each implementing country and measure type [i.e. the first time Australia adopted quarantine]. Columns 1 and 3 cover measures implemented from December 31 2019 to December 31 2020. Column 5 covers measures implemented from December 31 2019 to June 30 2020

About half of the adopted measures (48.9%) targeted all CTAs. Most of the rest targeted at least one WHO Member State (41.7%), while the remaining had no target CTA identified (8.7%), and thus were unmapped, or only targeted non-Member State CTAs (0.8%). Member States in the WHO European (EURO) region implemented the largest share of the measures in our dataset (38.9%). Similarly, Member States in the EURO region were the most likely to have been targeted by measures (28.6%). These findings are partially explained by the fact that there are many countries in the EURO region and then, when measures were applied to the EU, we captured many records in our dataset. WHO Western Pacific region (WPRO) Member States were the most targeted by the earliest measures (25.7%).

**Fig. 1:**
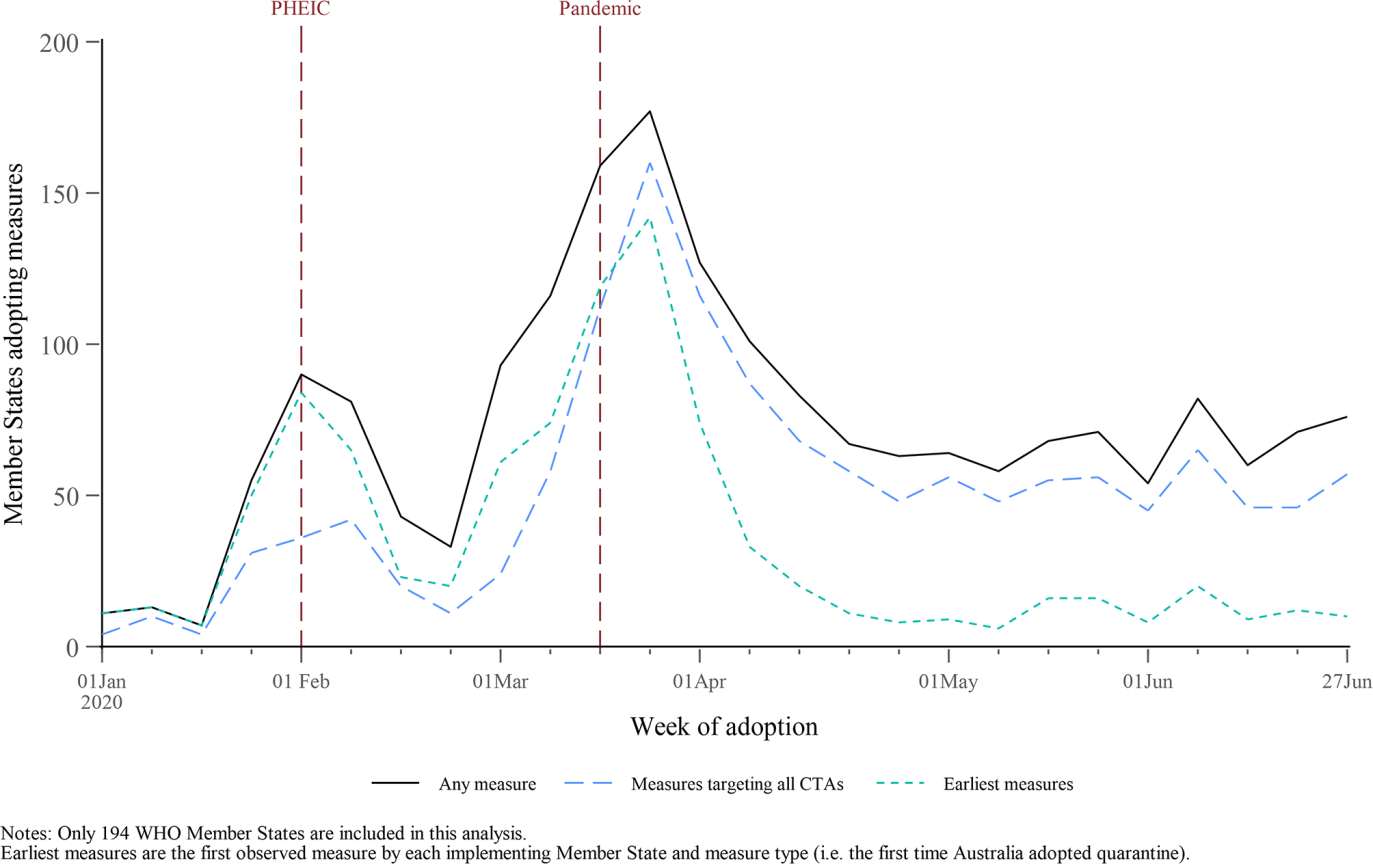
Number of Member States adopting measures, by week of adoption

Figure 1 reports the number of Member States adopting measures every week over the first six months of 2020. We see an initial rise in the number of states adopting measures in late January and early February, around the same time as the PHEIC declaration. After decreasing in late February, the number of Member States adopting measures rapidly increased during the first two weeks of March and peaked on the third week of March when 177 of 194 Member States adopted measures, which was soon after the WHO described the outbreak as a pandemic. This peak was much higher than the peak that coincided with the PHEIC declaration. By mid-April, the weekly number of states adopting measures dropped below 100, where it remained until the end of 2020 (see Appendix Fig. 2). The adoption of earliest measures fell to almost zero by May, further suggesting that most CTAs had implemented most of the types of measures they would maintain throughout the pandemic by this time. Measures targeting all CTAs, rather than targeted measures, became more common over time.

**Fig. 2:**
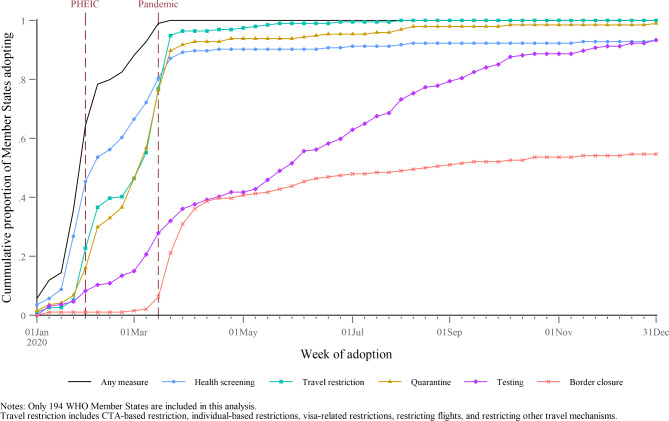
Cumulative proportion of Member States adopting measures, by measure type and week of adoption

Figure 2 presents the cumulative proportion of WHO Member States adopting specific types of measures in 2020. By the time WHO had declared the PHEIC, over 60% of Member States had already adopted some form of travel measure, typically health screenings and some form of travel restriction. In the weeks immediately following the PHEIC declaration, there were important shifts in the types of measures being adopted. The proportion of countries implementing travel restrictions or quarantine increased dramatically. By March 18, 2020, all Member States had adopted some form of travel measure. Testing of travelers also became more widely adopted over time, presumably as testing technology became increasingly available. The use of testing as a travel measure steadily increased throughout the first half of 2020 and was adopted by 60% of Member States by early July.

Before the pandemic declaration, only six Member States had implemented full border closure, which we defined as either no movement in or out by either land, sea, air, or all three modes of entry. Within a month of the pandemic declaration, nearly 35% of states went on to adopt this type of measure. There was, however, little subsequent take-up of the measure with it plateauing around 56% of Member States by the end of 2020.

**Fig. 3:**
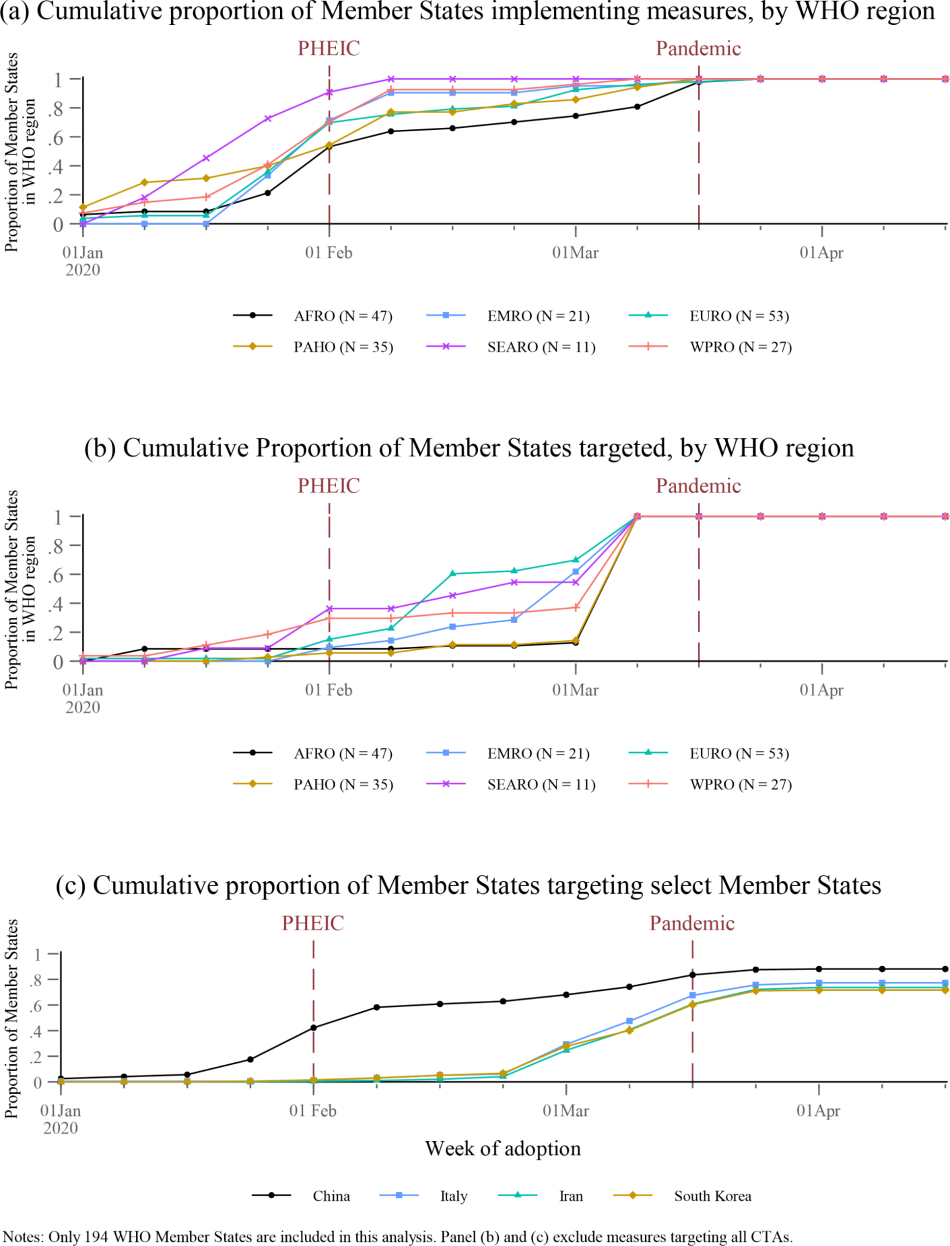
Cumulative proportion of Member States implementing and targeted by measures

Figure 3a depicts the cumulative proportion of Member States in each WHO region adopting measures in the first half of 2020. Proportionally, Member States in the Pan-American (PAHO) region were the earliest to adopt measures but were quickly outpaced by South East Asian (SEARO) Member States. Figure 3b depicts the cumulative proportion of Member States in each WHO region targeted by measures, excluding measures targeting all CTAs. Around the time of the PHEIC declaration, less than 40% of Member States in each region were targeted by travel measures, with states in WPRO and SEARO most likely to be targeted. By the declaration of a pandemic, every Member State had been targeted by at least one measure. In mid-February, the EURO region was the first to have half of its Member States targeted. Member States in the African (AFRO) and PAHO regions were the slowest to be targeted, remaining at less than 20% of states targeted through the end of February 2020. In the first week of March, all regions jumped to 100% of the Member States targeted.

Figure 3c depicts the cumulative proportion of Member States targeting four of the earliest targeted states. Excluding measures that targeted all CTAs, around 40% of Member States targeted China around the time the PHEIC was declared, increasing to more than 80% when the pandemic was declared. Meanwhile, Member States did not begin to target Italy, Iran, and South Korea until the last week of February. Within three weeks, the proportion of Member States targeting these countries increased from almost zero to about 60%, where it plateaued.

**Fig. 4:**
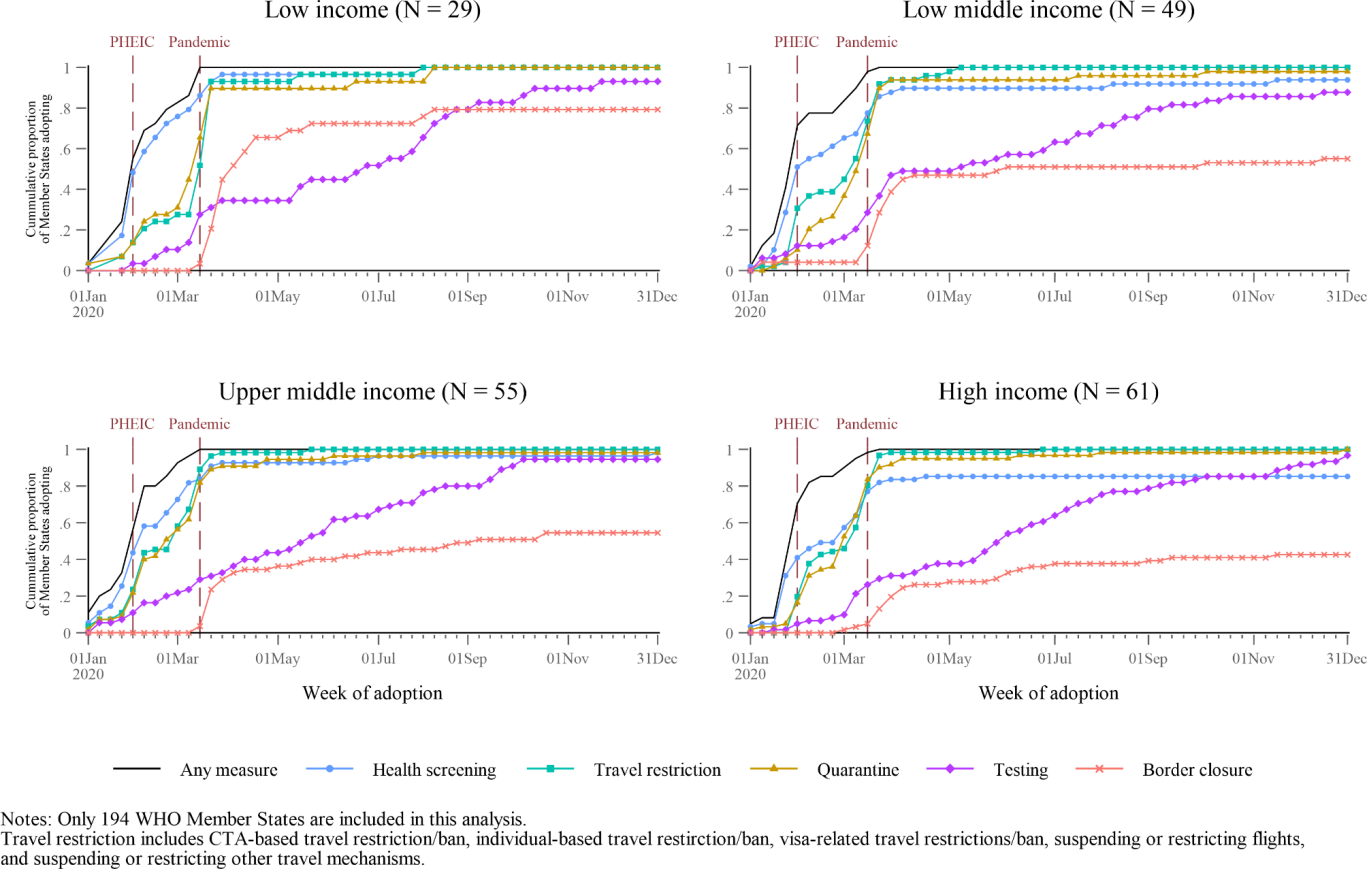
Cumulative proportion of Member States adopted measures, by income level, measure type, and week of adoption

Figure 4 shows the types of measures adopted by countries that differed by income level. Low-income countries initially relied almost exclusively on health screening. Following the announcement that COVID-19 met the criteria of a pandemic, these countries also dramatically scaled up quarantine and travel restrictions. Only about half of low-income countries had adopted any travel measure by the time the PHEIC had been declared. Soon after COVID-19 was declared a pandemic by WHO, almost 70% of low-income countries closed their borders, increasing to almost 80% by the end of 2020. It was not until September 2020 that nearly all low-income countries adopted some form of testing. In contrast, only about 40% of high-income countries ever adopted border closures, and instead were more likely to adopt a mix of measures early in 2020. Low-middle-income and upper-middle-income countries followed patterns that fall in between low- and high-income countries.

**Fig. 5:**
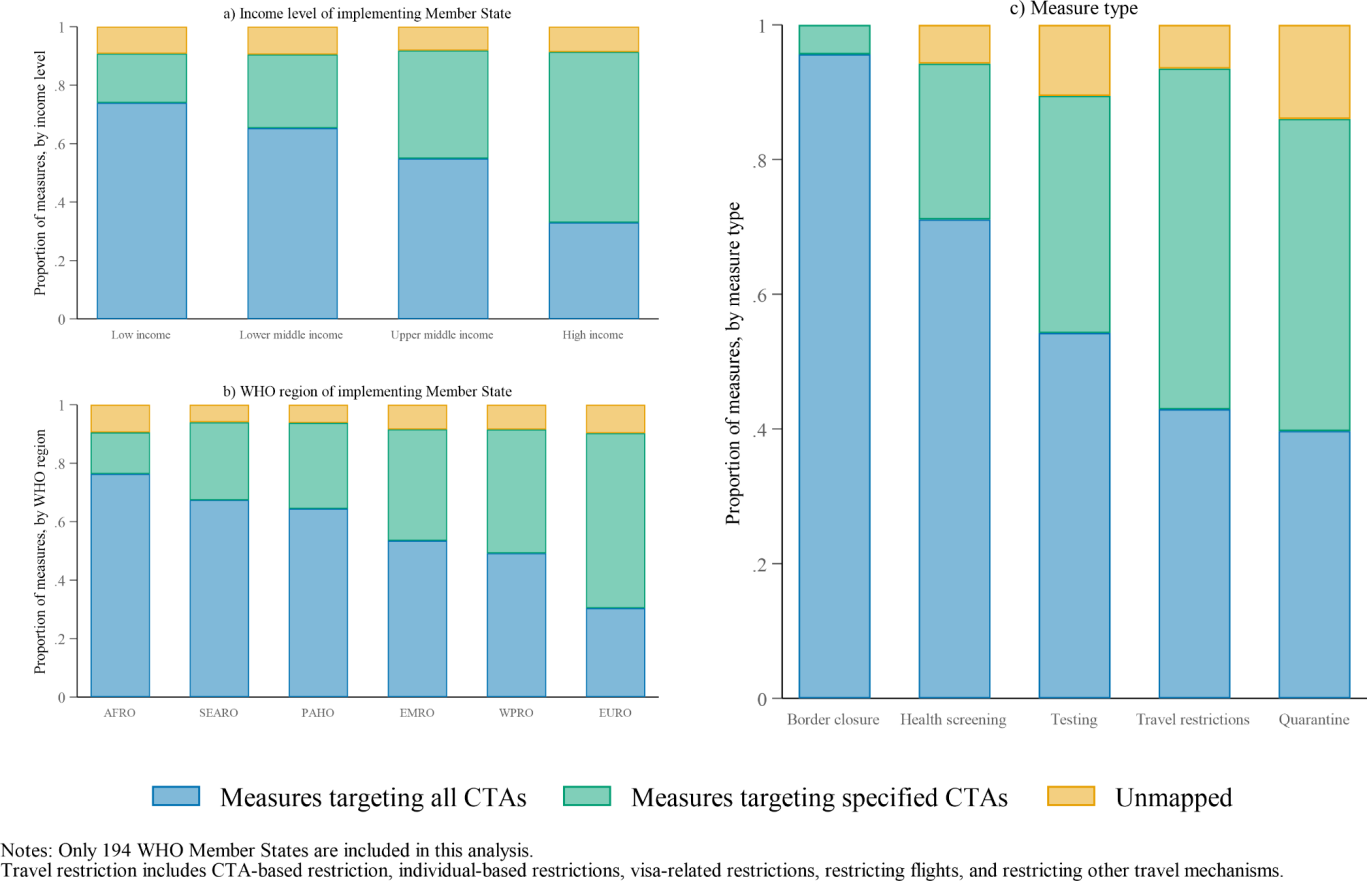
Proportion of Member States adopted measures targeting all CTAs or targeted measures, by income level, WHO region of implementing Member States, and measure type

Figure 5 shows the proportion of measures that are more universal in that they target all CTAs versus measures that target specific CTAs. In panel A, we see that the proportion of measures targeting all CTAs depends on the income level of the implementing state, with low-income countries relying the most on measures that target all countries and where almost 70% of adopted measures targeted all CTAs. In panel B, we see that across WHO regions, Member States based in the EURO region relied the least on measures that targeted all CTAs, while Member States in the AFRO region relied most heavily on these types of measures. This is perhaps not surprising given that most low-income countries are based in the AFRO region. In panel C, we see that border closure measures were the most likely to target all CTAs, followed by health screening. Quarantine was the least likely to be targeted at all CTAs.

## Discussion

Using a newly constructed dataset, we have identified previously unrecognized patterns in the adoption of international travel measures during the first year of the COVID-19 pandemic. First, the adoption of international travel measures was rapid, widespread, and largely inconsistent with WHO’s initial advice. Under the IHRs (2005), WHO is authorized to advise on the adoption of travel and trade measures at the time a PHEIC declaration is made but by the time the PHEIC declaration was made, 60% of Member States had already adopted some form of international travel measure. In late February 2020, WHO issued updated recommendations for international traffic, which were largely consistent with previous advice, namely they continued to recommend against the adoption of international travel or trade restrictions but the updated recommendations acknowledged that, in certain circumstances, such as settings with limited international connections, travel measures that significantly interfere with international traffic may be justified at the beginning of an outbreak to allow countries to gain time to rapidly implement effective preparedness and response measures. Around the same time as these updated recommendations were issued, a second wave of adoption of international travel measures by Member States began. Health screening, which does not typically “significantly interfere” with international travel [6], was initially the most widely adopted measure. However, by the time the PHEIC was declared, over 68% of Member States adopting measures had already adopted measures beyond health screening, and nearly all (94%) had done so by the time COVID-19 was labelled a pandemic (calculation not shown). In short, this study provides evidence that the adoption of travel measures by Member States became less consistent with WHO’s guidance under the IHRs following the PHEIC declaration.

Second, and similar to a previous study [6], we found that the pandemic announcement was associated with the adoption of more restrictive and more universally applied measures than the PHEIC declaration. Although we cannot infer this association was causal, since there was also increased media attention to COVID-19, more countries reporting domestic cases, and a rapid increase in the number of cases reported globally during the same period as the pandemic announcement, Worsnop et al. (2022) found that the timing of the adoption of international travel measures was independently associated with the timing of the pandemic declaration after controlling for media attention and the number of reported incident cases [6]. Thus, following the pandemic announcement, our data suggests Member States were even less consistent with WHO recommendations.

It is not well understood why so many countries did not follow WHO recommendations against the use of travel restrictions following the PHEIC declaration, and even less is known about what changed between late January and mid-March for even more Member States to adopt measures. There are several possible, and interrelated, explanations: changes in the perception of the effectiveness of measures, political factors, increased awareness of the pandemic after WHO characterized COVID-19 as such, or shifting WHO guidance on the use of international travel measures [5]. Interestingly, one study from the United States found that public opinion was generally supportive of the use of international travel restrictions during the pandemic, but that people were less supportive of the use of these measures once they understood WHO’s position on the use of these measures [20]. At the 2024 World Health Assembly, a number of revisions to the IHRs were adopted by Member States, most notably that WHO can now make two types of declarations: a PHEIC and a new pandemic emergency. Other relevant changes include strengthening a dispute resolution process regarding international travel and trade restrictions and the creation of a new committee on IHR implementation. The proposed amendments would come into force around May 2025, unless Member States opt-out. It will be interesting to see if these revisions will change Member States proclivity to adopt international travel measures during future outbreaks of pandemic potential. More research is needed to better understand why more governments adopted international travel measures over time, and what role if any, WHO guidance or the IHR played.

Third, there were important changes in the types of measures adopted during the first year of the COVID-19 pandemic. Initially, most measures adopted were health screening (e.g., temperature checks), which studies have generally concluded were of low benefit in terms of identifying potentially infected travelers [9]. Over time the types of measures introduced broadened to include border closures, quarantine, and eventually testing, perhaps once the technology to do so became more widely available. Governments were also much more likely to adopt measures targeting all CTAs over time. This is unsurprising. While the focus was firmly on China in January and February 2020, as cases were identified worldwide and COVID-19 was declared a pandemic, countries adopted measures targeting all CTAs. What is surprising is that many countries did not universally apply measures such as quarantine and testing to all CTAs despite such measures being more effective for achieving public health goals when applied to all travelers [9].

Fourth, the types of measures adopted, and the timing of when they were adopted, varied by the income level of countries. Initially, lower-income countries relied more heavily on health screening and were the most likely to adopt full border closure. They were also slower to scale up testing compared to higher-income countries. There might be many reasons why these countries pursued a different approach. While it is unclear if the public health benefits of implementing these measures outweighed their costs, studies have generally concluded that measures adopted earlier and more universally targeted were more likely to be effective for public health goals [9]. If a lack of resources or access to technology limited their ability to implement measures with greater potential public health benefits at an earlier date, then the travel measure needs of low-income countries should be further considered in pandemic preparedness planning efforts. It might also explain why low-income countries were more likely to rely on applying universal border closure measures, perhaps as a last resort when more targeted measures may require greater capacity to implement.

While our study helps to fill some important data gaps in our understanding of what, when and how international travel measures were adopted, we acknowledge several important limitations to this study. First, the PHSM dataset was largely developed using media sources, rather than official government data, as many governments did not officially report their use of travel measures to WHO (and government reports to WHO are not publicly available). This limited our ability to verify the information provided by available sources. It is also likely that there may have been systematic under-reporting of some types of measures or from some types of CTAs. For example, it is likely that data from smaller CTAs were less likely to be reported by media sources and thus these jurisdictions might be under-represented in our dataset. Second, and partially because we were required to rely mainly on media-sourced data, there are some very important gaps in our dataset. For example, we generally reported the earliest measure in our dataset for each country and measure type. If a measure was observed in our dataset, we were generally confident that it had been adopted by a given CTA, but we cannot confirm that the measure in our dataset was always the first measure introduced in each country. Third, it would be very useful to know when countries stopped using or removed such measures. Once again, most Member States did not officially notify WHO of this information or issue public communication on a routine basis during the pandemic. The removal of measures was also not as consistently reported in the media. It is therefore not possible for us to report on when these measures ended. Fourth, despite the substantial efforts to improve data quality and coding procedures, inconsistencies in data coding were unavoidable. This issue was prominent for measure status (see Appendix Table 5), which we ultimately decided not to include in our analyses. Finally, the varied language used to describe measures by governments and the media did not always allow for accurate coding of the specific type of measure adopted. The use of the term “border closure” or similar rhetoric was especially common but, upon closer scrutiny, many reports of border closures did not comply with the more stringent definition applied in this study.

## Conclusion

The adoption of international travel measures was widespread during the COVID-19 pandemic. All WHO Member States adopted some form of international travel measure in the weeks following WHO’s declaration that COVID-19 constituted a pandemic. Many Member States adopted travel restrictions despite WHO recommendations against their use. The types of measures adopted during the pandemic were more diverse and implemented over a longer time than during previous PHEICs, such as the Ebola virus outbreak in West Africa (2014–2016) and H1N1 influenza pandemic (2009). While evidence reviews report that the early adoption and universal application of certain types of travel measures (e.g., quarantine in combination with testing) may have been warranted from a public health perspective in response to COVID-19, there remains limited understanding of whether, what, when and how such measures should be used in future outbreaks and pandemic events.

From the perspective of the IHRs, during a PHEIC WHO’s role is to issue Temporary Recommendations on the use of international travel measures to achieve the IHR’s dual goals of protecting public health while minimizing unnecessary interference with international traffic. Although the effectiveness of travel measures is still not fully understood, this descriptive analysis provides important insights into which measures were adopted, when they were applied, adopted by and targeted at which countries, to advance our understanding for future practice. More detailed research is needed to understand why governments adopted measures in these ways and, importantly, how governments might better cooperate to enhance the benefits from their use and/or reduce their adverse social and economic impacts.

Finally, our study demonstrates the need for standardized terminology and typology of international travel measures, as well as the establishment of a global data reporting system, ahead of future outbreaks. This will enable essential research to better inform decision-making by governments, the travel sector, and travelers.

## Electronic supplementary material

Below is the link to the electronic supplementary material.

Supplementary Material 1

## Data Availability

Dataset is based on publicly available data. Data can be shared to others subject to valid request.
