# Supplementary material for: The adoption of international travel measures during the first year of the COVID-19 pandemic: a descriptive analysis
Source: Global Health. 2024 Oct 4;20:72. doi: 10.1186/s12992-024-01071-7 (PMC11451053; doi:10.1186/s12992-024-01071-7)
Supplement: Supplementary file 1 — Supplementary Material 1 [file 12992_2024_1071_MOESM1_ESM.docx]

**The adoption of International Travel Measures During the**

**First Year of the COVID-19 Pandemic: a descriptive analysis**

**Appendix**

**Table of Contents**

[1. The underlying dataset 1](#_Toc166773859)

[2. Taxonomy of measures 4](#_Toc166773860)

[Criteria for inclusion 4](#_Toc166773861)

[Data schema 4](#_Toc166773862)

[3. The data coding process 12](#_Toc166773863)

[Data preparation 12](#_Toc166773864)

[Phase One: Data re-coding 12](#_Toc166773865)

[Phase Two: Data review 13](#_Toc166773866)

[Phase Three: Data cleaning 14](#_Toc166773867)

[4. Analysis 14](#_Toc166773868)

[Limitations and usage notes 33](#_Toc166773869)

[Data & Code Availability 34](#_Toc166773870)

[5. Additional Tables and Figures 34](#_Toc166773871)

[6. References 38](#_Toc166773872)

## 1. The underlying dataset

At the outset of this project, we conducted a review and comparison of the major COVID-19 policy tracker datasets (see Cheng et al 2022 for a review of the available trackers), which allowed us to identify four major databases with good coverage of international travel measures: the Public Health and Social Measures (PSHM)^[[1]](#footnote-1)^ dataset that was coordinated by WHO , Coronanet^2^, and the Oxford Covid-19 Government Response Tracker (OXCGRT) ^3^, and COVID Border Accountability Project (COBAP) ^4^. Table 1 summarizes these databases.

*Table 1: Databases of international travel measures*

|  | PHSM | Coronanet | OxCGRT | COBAP |
| --- | --- | --- | --- | --- |
| Focus | Public health and social measures, not restricted to government response | Government responses to Covid-19 | Government responses to Covid-19 | International border closures during 2020 |
| Data Source | Combination of five trackers (see Table 2) | Hand-coded from publicly available government sources | Manual searches of publicly available news articles, government press releases and briefings. | Manual search for official government sources, with limited use of major news outlets |
| Implementing CTAs | 234 | 210 | 184 | 246 (235 having measures) |
| Targeted CTAs | Partial (Listed by name in some cases) | Yes | No | Partial (Listed by name in most cases) |
| Types of international travel measures captured | Entry screening and isolation or quarantine, suspending or restricting international flights, closing international land borders, restricting entry, providing travel advice or warning, suspending or restricting international ferries or ships, restricting exit, exit screening and isolation or quarantine, restricting visas | External border restrictions with subcategories travel history form, visa restriction, health screenings, health certificates, visa extensions, and other. | Containment and closure subcategory restrictions on international movement. | Complete border closure, with subcategories essentials-only exception, citizenship exception, specific countries exception, workers exception.  Partial border closure with subcategories visa bans, citizenship bans, travel history bans, border closure to land, sea, or air but not all three) |
| Start date of data | December 31, 2019 | December 31, 2019 | January 1, 2020 | January 1, 2020 with the first datapoint on January 22, 2020 |
| End date of data | After December 31, 2020 | After December 31, 2020 | After December 31, 2020 | December 31, 2020 |
| End date of measures | Partial | Partial | No | Yes |

Based on this review, we concluded that WHO’s PSHM^1^ had collected the most comprehensive data on international travel measures and thus it was selected for our. PSHM had collated data from six trackers, which are ACAPS Covid-19 Government Measures Dataset^[[2]](#footnote-2)^, Johns Hopkins University (JHU) Health Intervention Tracking for Covid-19 (HIT-COVID)^[[3]](#footnote-3)^, Oxford Covid-19 Government Response Tracker (OxCGRT)^[[4]](#footnote-4)^, US Centres for Disease Control and Prevention International Task Force (CDC-ITF) Mitigation Measures Dataset, and WHO EURO’s PHSM dataset^[[5]](#footnote-5)^. Table 2 provides a brief description of the data sources of the five trackers.

After completing our data coding, we also learned of another dataset, the Global Mobility Restrictions Overview, which was collected by the International Organization on Migration (IOM) in collaboration with the International Air Transport Association (IATA) (see details on how to access in our reference list). While it collected data on thousands of international travel measures adopted during the pandemic, it had limited data in early 2020, an important period of interest to us in this research project, and it only collected data on a narrower set of measures. Since our goal was to analyze the full range of measures adopted during the pandemic, this dataset was also not useful to us for these analyses. However, a strength of this dataset compared to the other datasets, is that it maintained data collection through the end of 2022.

*Table 2 The data sources of the six underlying datasets of PHSM*

| Tracker | Data source |
| --- | --- |
| ACAPS | Web search, governments (official sites, embassies), media, United Nations agencies and other organization. Priority is given to official/governmental sources. |
| HIT-COVID | Official government sources were preferred, but other sources were permitted when official sources were unavailable. |
| OxCGRT | Publicly available sources such as government press releases and briefings, international organization reports and trusted news articles.^[[6]](#footnote-6)^ |
| CDC-ITF | Publicly available sources, including news media, ministry of health websites, and scientific publications. |
| WHO EURO PHSM | Government websites, WHO country office and emergency hub communication, media sources, thematic webpages, and PHSM publicly available databases.  Information from Member States and notifications of national International Health Regulations (IHR) focal points sent via the WHO regional IHR contact point or through the European Early Warning and Response System was also included. |

We reviewed the data and methodology used to construct the PHSM database to understand its limitations and the modifications we would need to make to meet our research objectives. The five trackers were converged using standardized variables and WHO taxonomy. Each row in the PHSM database represents a CTA implementing a measure. The resulting dataset was then cleaned: duplicates were removed, PHSM coding was validated and verified, and additional variables were coded for each entry.^[[7]](#footnote-7)^

We noted several limitations of the PHSM database on which we could improve. First, it does not capture data on which CTAs were targeted by a travel measure. Second, it uses a coding scheme for the travel measures that we felt did not fully capture the full spectrum of travel measures introduced by CTAs during the pandemic. Third, we conducted a quality check of a random sample of 500 measures to verify the accuracy of the data contained in the database. Common data issues identified were 1) wrong start date; 2) miscoded measure type or subtype; 3) duplicate entries; 4) national measures for federal entities incorrectly coded as sub-national measures; 5) measures referencing removed measures; 6) inaccessible or expired source links; and 7) measure descriptions not found in the provided link. As such, we believed the data would need to be further verified before use. To address these limitations, we developed our own taxonomy of travel measures and mapped the measures in the PHSM to those in our coding scheme.

## 2. Taxonomy of measures

### Criteria for inclusion

We define a “measure” as an action taken by a government institution to control movement of people across two or more international CTA with the stated intent of preventing, controlling, or mitigating the impact of Covid-19. Each row in our database represents a measure of unique implementing CTA, measure type, measure subtype, week of implementation, measure type, measure subtype, and target CTA.

This definition excluded the following:

- Actions implemented for reasons other than public health.
- Actions that did not affect international travel but only limited movements within a CTA.
- Decisions made by a non-government institution (e.g. a private company).
- Actions where the source suggests that a measure is under planning.
- Actions associated with international trade, travel bubbles, and zoning.

### Data schema

Our database contains the following dimensions for each record:

- Implementing country/territory/area (CTA)
- The 3-digit ISO code of the implementing CTA
- WHO region of Member States (if applicable)
- The start date of the measure
- Source link
- Whether the measure is applied to the whole implementing CTA or a sub-region of the CTA
- The sub-CTA units imposing the measure (if applicable)
- Whether the measure targets the whole CTA, a sub-region of a CTA, or both.
- The measure type
- The measure subtype (if applicable)
- The measure status, or whether a measure was new, or the type of change made to a previous record
- The following measure (if applicable)
- The previous measure (if applicable)
- The geographical target of the measure (including country, region, province, city, and other geographic locations)
- Whether the measure involved Covid-19 vaccine, either by requiring the provision of vaccination records or exempting vaccinated travelers from the measure
- A textual description of the measure

Figure 1 Taxonomy of measure types and sub-types


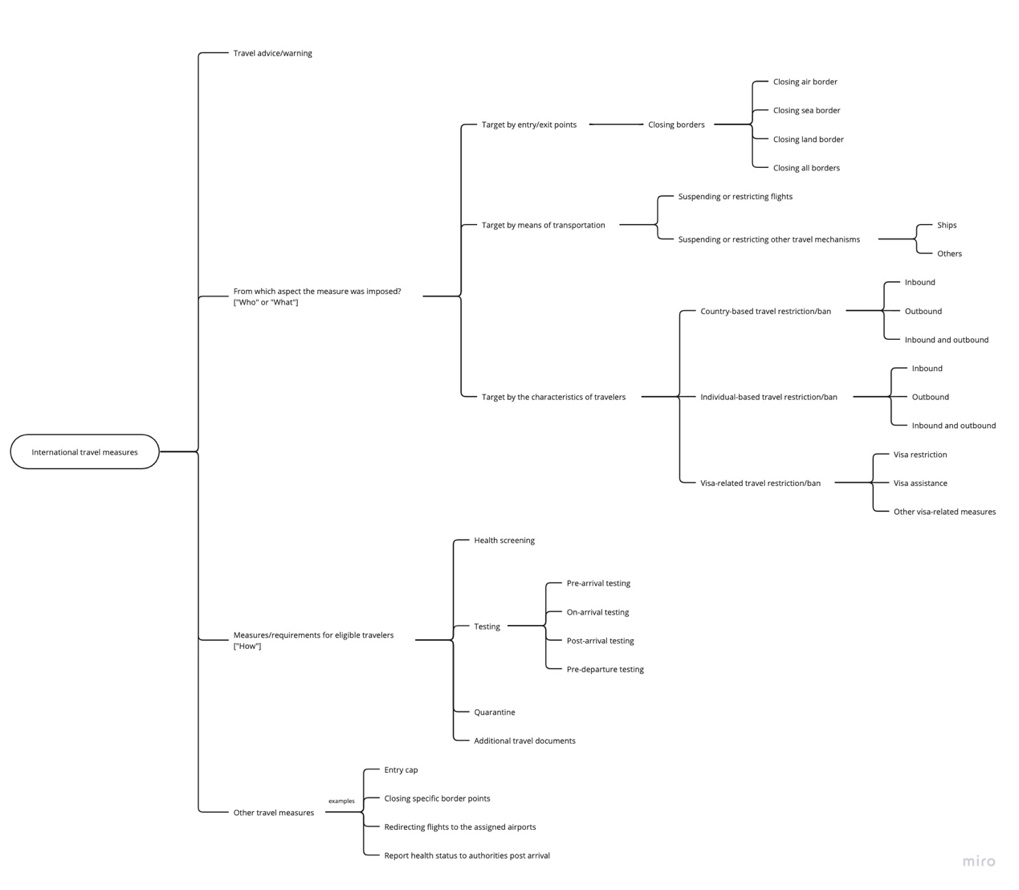


*Table 3 Measure type*

| Measure Type | Definition | Example |
| --- | --- | --- |
| Providing travel advice or warning | Health advice or warnings on CTA-level transmission of Covid-19 to guide individual decisions on travel. | The Angolan authorities has advised against Angolan citizens to travel to China where Covid-19 outbreak is ongoing. |
| Health screening | Evaluation of the health or exposure status of travelers entering or exiting a CTA. Exposure may be defined as arriving from an affected CTA. Excludes testing and additional travel documents. This usually refers to temperature check and monitoring symptoms of travelers. | Everyone arriving in Afghanistan needs to have their temperature checked at the airport. If your temperature is high, the government will advise you to quarantine and will offer you medical attention at two hospitals in Kabul. |
| Quarantine | Separating and limiting the movement of international travelers who have or may have been exposed to Covid-19. Includes both facility quarantine and home quarantine. | Brazil: Home quarantine is mandatory for national citizens, resident foreigners, and members of the diplomatic corps coming from outside Brazil.  Greece: If you originate from an airport on the EASA affected area list, then you will be tested upon arrival. An overnight stay at a designated hotel is required. If the test is negative, the passenger self-quarantines for 7 days. If the test is positive, the passenger is quarantined under supervision for 14 days. |
| Testing | Any measures related to Covid-19 test(s) which international travelers would experience before, during, and/or after their arrival to their destination. |  |
| Closing borders | Complete closure of land, sea, or air borders, without specifying targeted CTAs/population groups/travel routes. | Peru: Air, sea and land borders are closed, except to trade and goods. |
| Suspending or restricting flights | Stopping arrival of international flights, restricting the origin or number of flights, rescheduling of flights, or closing airports, without specifying targeted routes. | On Thursday, March 12, the Peruvian government announced that flights to and from all destinations in Europe and Asia will be banned starting on Friday, March 13, in efforts to curb the ongoing spread of Covid-19. |
| Suspending or restricting other travel mechanisms | Stopping, restricting, or rescheduling non-airline arrival through (e.g., ferries, ships, trains). | Greece: Arrivals by sea by private yachts, cruise ships, ferry ships and any other professional tourist ships, regardless of flag or destination abroad, as well as the disembarkation of passengers from these boats in any way is prohibited. |
| Individual-based travel restriction/ban | Restricting/suspending the entry/exit of travelers based on characteristics (e.g., nationality, residency, occupation). | People who are not Australians, permanent residents or their families AND who have been in China cannot enter Australia.  Only Fijian citizens and residents are permitted to enter Fiji at this time. People from any other countries cannot enter unless they have specific grounds for exemption.  On February 14, the Philippines lifted a restriction that denied entry to individuals who had been to Taiwan 14 days before traveling to the Philippines. |
| CTA-based travel restriction/ban | Restricting/suspending international travel between a CTA and one or more foreign CTA. | Australia puts travel ban on South Korea.  Kurdistan has banned travel with Iraq China, Iran, Bahrain, Kuwait, Japan, South Korea, Thailand, Singapore, and Italy. |
| Visa-related travel restriction/ban | Suspending or restricting the issuance of visa (or specific types of visas) for international travelers.  OR  Providing assistance for visa holders whose international travel plans are interrupted due to the COVID-19 pandemic. | All Gabon diplomatic missions abroad are prohibited from issuing entry visas to Gabon.  Foreign nationals situated in Iceland, who are unable to return to their home countries due to travel restrictions, quarantine or isolation, are allowed to stay in Iceland without a residence permit or visa until 10 September 2020, according to an announcement of the Ministry of Justice. |
| Additional travel documents | Imposing additional requirements on documents for international travel, or changing the issuance/validity of travel documents, which include, but are not limited to, health declaration form/questionnaire, entry approval letter, health insurance (excluding pre-arrival testing certificate). | Thailand requires all travelers purchasing health insurance that must be covered in Thailand at least USD100,000.  Timor-Leste: Individuals of foreign nationality wishing to enter national territory via the land border must apply for registration and ask for entry permit at consular offices. |
| Other travel measures | Any other measures aiming at regulating international travel which do not meet the definition of the above-listed measure types. | Only 5,000 people per week are allowed to enter Greece on flights from Israel arriving to all airports in the country. |
|  |  |  |

*Table 4 Measure subtype*

| Measure Subtype | Parent Measure Type | Definition | Example |
| --- | --- | --- | --- |
| Pre-arrival testing | Testing | Requiring inbound travelers to provide a test result of a specified Covid-19 test conducted within a certain timeframe. | All international travelers are required to present a negative Covid-19 test certificate dated no more than 72 hours prior to arrival in Chad. |
| On-arrival testing | Testing | Conducting Covid-19 test for travelers upon their arrival. | Eswatini: Incoming travelers who do not present a valid negative result, and those presenting with symptoms regardless of valid negative results, will be required to do a Covid-19 test at the point of entry at the traveler's own expense. |
| Post-arrival testing | Testing | Conducting Covid-19 test for travelers in the days or weeks after their arrival. | After the seventh day of quarantine, passengers of UK flights will have to take a new PCR test. If the test is positive the quarantine will be in force for 14 days. If the test is negative the quarantine ends. |
| Pre-departure testing | Testing | Requiring outbound travelers to provide a test result of a specified Covid-19 test conducted within a certain timeframe. | All passengers departing from Sri Lanka will be required to present a negative RT-PCR test for Covid-19. The test must be taken within the 72 hours preceding the departure flight. |
| Visa restriction | Visa-related travel restriction/ban | Suspension of visas or restriction of issuing visa for specific travelers (e.g., travelers originating from COVID-affected countries). | The Myanmar Government temporarily suspends issuance of Visa on Arrival and e-visa for all countries until 30 April 2020. |
| Visa assistance | Visa-related travel restriction/ban | Providing assistance for visa holders or applicants whose international travel arrangements are affected by the Covid-19 pandemic. Includes measures to maintain normal issuance of visas during the pandemic. | Guatemalan immigration office announced on April 6, 2020 that non-Guatemalans who entered Guatemala legally using a tourist visa, but who are unable to depart due to the travel restrictions put in place by the Guatemalan government in response to the Covid-19 pandemic, will be allowed to remain in Guatemala in legal status until the travel restrictions are lifted, even if their 90-day authorization to remain in Guatemala has lapsed at that time. |
| Outbound | CTA-based travel restriction/ban | Restricting/suspending outbound travel to a foreign CTA. |  |
| Inbound | CTA-based travel restriction/ban | Restricting/suspending inbound travel from a foreign CTA. |  |
| Outbound | Individual-based travel restriction/ban | Restricting/suspending the exit of travelers based on characteristics (e.g., nationality, residency, occupation). |  |
| Inbound | Individual-based travel restriction/ban | Restricting/suspending the entry of travelers based on characteristics (e.g., nationality, residency, occupation). |  |
| Closing borders: All borders | Closing borders | Complete closure of land, sea, and air borders, without specifying targeted CTAs/population groups/travel routes. |  |
| Closing borders: Air borders | Closing borders | Complete closure of air borders, without specifying targeted CTAs/population groups/travel routes. |  |
| Closing borders: Land borders | Closing borders | Complete closure of land borders, without specifying targeted CTAs/population groups/travel routes. |  |
| Closing borders: Sea borders | Closing borders | Complete closure of sea borders, without specifying targeted CTAs/population groups/travel routes. |  |

*Table 5 Measure status*

| **Measure status** | **Description** | **Example** |
| --- | --- | --- |
| Introducing | The introduction of a new measure, including  1) a new measure type  2) a new measure subtype  3) a new geographical target (location/population/direction) | Greece changes the scheme of Covid-19 testing on foreign Travellers arriving in the country. Random testing according to “green” and “red” countries is over, targeted testing is in. Travellers will receive the barcode after they have filled out the Passenger Locator Form. They will show the barcode on printed form or on their mobile phones upon their arrival in the country’s airports. An algorithm that will calculate which travellers are most likely to be carriers of the coronavirus. |
| Strengthening | Changes to an existing measure that makes it more restrictive for travelers. This usually includes:  1) More geographical locations or population groups are affected by the measure  2) The duration for quarantine is extended  3) More restrictions on testing requirements (# of tests, type of tests, time frame for pre-arrival testing)  4) More borders (air/sea/land/all) are closed  5) Extra requirements on travel documents | - Country A,B,C are added to the travel ban list. - Abu Dhabi health authorities announced that anyone entering Abu Dhabi emirate must take **an additional** PCR test on the sixth consecutive day in the emirate, besides of the pre-arrival test they have to take before boarding a plane (which was introduced before). |
| Extending | The end date of a measure is extended without changing other elements of a measure. | The suspension of all passenger flights to and from Greece to Italy, Spain, the United Kingdom and the Netherlands is extended until 31 May. |
| Easing | The relaxation/reduction of an existing measure. This usually includes:  1) Fewer geographical locations or populations are affected by the measure  2) The duration for quarantine is shortened  3) Fewer restrictions on testing requirements (# of tests, type of tests, time frame for pre-arrival testing)  4) Fewer borders (air/sea/land/all) are closed  5) Reducing the requirements on certain travel documents | - Crossing the Slovene-Italian border outside the checkpoints will be allowed, but only for Slovenian and Italian citizens. (the border closure is phased out, but is not ended/finished) - The Swedish Ministry of Foreign Affairs department removes the advisory against non-essential travel to Andorra, Poland and Germany after the 15th of July. (Although the warning is removed for three countries, there are still advisory against other countries) - Commercial flights between Suriname and the Netherlands, as well as highly regulated pre-arranged regional flights, are permitted. Nonetheless, the authorities will determine the number of passengers per flight per week. |
| Modifying | Make changes to an existing measure that cannot be categorized as strengthening, easing, or extending. | - Specifying or changing approved testing facilities or test types - Stipulating the language of additional travel documents. |
| Finishing | The end of a measure type and subtype. | - Armenia lifted ban on the entry of foreign citizens. - As of 22-May, all border crossings are opened without restrictions. - The Government of the United Republic of Tanzania has opened its airspace to restore its conventional transportation involving commercial airlines and passengers to the various country. |
| Re-introducing | Introducing a measure again after it is finished. | - The following countries are again covered by the travel quarantine become red countries again: France, the Czech Republic, Switzerland, Monaco. - On 15 July, a travel advisory against non-essential travels will be reintroduced to Switzerland until 29 July. |

## 3. The data coding process

### Data preparation

A coding protocol was developed, including a pilot test and subsequent adjustments based on coder feedback. Using Python, a research assistant (RA) then extracted the relevant variables from the PHSM dataset, including all records categorized as “international travel measures”. They grouped records from each CTA in a separate Excel file and added variables and to be manually coded.

### Phase One: Data re-coding

The initial round of data coding was conducted from May to September 2021. All CTAs were grouped into four batches based on the alphabetical order of the first letter and were assigned to coders at random. For each record of assigned CTAs, coders performed the following steps:

1. Indicated if the measure did not meet the inclusion criteria based on the description provided by PHSM and the link in the “SOURCE” column.
2. Indicated if the measure was a duplicate entry.
3. Checked if the link in the “SOURCE” column worked. Any issue associated with the source link were recorded in “LINK_NOTE”. If the coders found a source for a measure with a missing source or an alternative source with better quality, the link was entered into the “OTHER_NOTE” column^[[8]](#footnote-8)^.
4. Coded “TARGET_COUNTRY”, “TARGET_PROVINCE”, “TARGET_CITY”, “TARGET_REGION”, and “TARGET_OTHER_GEO” using information provided in the description, the associated link, and PHSM “LOCATIONS” variable. Measures were determined to target “all countries” if no target population was specified or if the measure type was “closing borders”.
5. Coded other variables based on the measure description, including "DECISION_MAKER", “MEASURE_TYPE”, “MEASURE_SUBTYPE”
6. Coded “UPDATE_TYPE” (later renamed “MEASURE_STATUS” for clarity) based on “MEASURE_STAGE” and the measure description.
7. If a judgment could not be made based on all available information, the coder would input “cannot decide” or similar texts indicating the challenges occurred into the relevant “MEASURE_ NOTE”, “UPDATE_NOTE”, “TARGET_NOTE”, or “OTHER_NOTE” columns.

There are two scenarios where new records would be created. First, over the course of verifying existing data, coders could identify a previously unrecorded international travel measure.^[[9]](#footnote-9)^ Second, as we expanded on the measure types and subtypes used in the PHSM, some measures in the source dataset contained multiple measure types and subtypes under our coding scheme. When this occurred, we used the original record and measure description provided by the PHSM dataset to code these measures into separate rows with unique identifiers.

To ensure that each measure was coded in a consistent manner by different coders, we adopted a ‘learn-by-doing’ approach, which involved regular training sessions and discussion amongst coders. For each coder, the lead coder reviewed whether variables of a random selection of entries were coded correctly and provided individual feedback. The Principal Investigator (PI) was consulted when the lead coder could not find the best solution to specific problem. The final decision was made upon agreement by the two persons.

### Phase Two: Data review

Measures flagged with one or more issues in Phase One were extracted for further review when (1) “Cannot decide"/"Complicated"/"Subtype not specified”^[[10]](#footnote-10)^ was entered in “MEASURE_NOTE” or (2) the “TARGET_NOTE”, “UPDATE_NOTE”, or “OTHER_NOTE” columns were not blank. A decision was made by a second coder, based on a discussion with all coders, and/or by the lead coder. Common resolutions included:

- Deciding on a measure type or subtype.
- Implementing data corrections indicated during Phase One (e.g. new source link, start date information is wrong).
- Searching for alternative sources. In the case of an uninformative link and comments, the corresponding text of a credible link was input in “MEASURE_DESCRIPTION”. In the case of unspecified or unconfirmed targets, CTA names confirmed by the alternative source were added to “MEASURE_DESCRIPTION”. Other variables may have been updated based on the new link.

The column REVIEW_NOTE was used to indicate actions taken during data review. At the end of Phase Two, all records with a non-blank REVIEW_NOTE were reviewed by the lead coder and merged with the records not flagged for Phase Two.

To verify the quality of records not flagged during Phase One, 5% of measures from the dataset were randomly selected for further verification. For each selected record, a second coder used the methodology described in Phase One to create a new record for cross-verification. The leader of the coding team then compared the original and the new record. A final decision was made upon discussion of all coders.

### Phase Three: Data cleaning

In the third phase, we focused on removing major errors and inconsistencies in the dataset. During this stage, we

1. Excluded measures imposed after April 20, 2021, entries without any information other than “WHO_ID”, measures imposed by non-government decision makers (e.g., airlines);, duplicate entries, and measures flagged as “Not a measure” under the “MEASURE_TYPE” column.
2. Input missing data by double checking the existing description of a measure and/or searching for alternative sources.
3. Extracted all entries without any target geographic locations, verified the input data, and manually coded any missing data.
4. Ensured that each entry has a unique identifier and decided on a case-by-base basis whether the entries with the same identifier should be removed or assigned a new identifier.
5. Identified and corrected mismatches in measure type and measure subtype.
6. Fixed structural errors such as variable names, variable data types, spelling mistakes, misalignment in capitalization, excessive blank space between text, etc.
7. Removed resolved notes of issues and questions inputted by coders.
8. Removed temporary variables created for Phase One and Phase Two

During the coding process, WHO regularly updated the PHSM dataset. Our database was coded using the April 20, 2021 version. The lead coder compared this version with the October 4, 2021 version and noted that October 4th version had fewer “international travel measures” and “national-level” measures for the same time period as the April 20 version. This indicated that the PHSM team had deleted duplicate entries or removed entries that were not international travel measures. After this analysis, we extracted 297 additional “international travel measures” from the December 14, 2021 version of the PHSM dataset. These additional measures did not appear in the April 20^th^ version and had start dates before our cut-off date of April 20, 2021. The lead coder followed the standard procedure described in Phase One for each of the 297 records.

## 4. Analysis

Analysis was done using Stata 18. Starting from Phase Three data, modifications were made to facilitate data analysis. This included standardization of target CTA and region to improve keyword searches used for defining target Booleans (see below) and for Table 7.

We also made some data corrections of errors identified during the analysis process. Most of these were identified from spot checks of records that provided target descriptions other than ISO codes, and from abnormalities in figures and graphs. For instance, we added or removed target CTAs based on measure description and/or links of about 30 records. Additionally, two records had their start date corrected, one record had its measure status changed from extension to easing, and three CTAs that are not WHO Member-States had the WHO region removed.

Our database includes 237 implementing CTAs extracted from the PHSM dataset. We defined 252 CTAs as potential targets using ISO 3166-1 alpha-3 codes plus four CTAs that were included as implementing in the PHSM dataset but do not have ISO-3 codes. Table 6 summarizes the implementing and targeted CTAs.

*Table 6: Implementing and Targeted CTAs*

|  | **ISO-3** | **CTA Name** | **Implementing CTA** | **Target CTA** | **WHO Member State** | **WHO region** | **Income Classification** |
| --- | --- | --- | --- | --- | --- | --- | --- |
| 1 | ABW | Aruba | Y | Y | N |  | High income |
| 2 | AFG | Afghanistan | Y | Y | Y | EMRO | Low income |
| 3 | AGO | Angola | Y | Y | Y | AFRO | Lower middle income |
| 4 | AIA | Anguilla | Y | Y | N |  |  |
| 5 | ALA | Åland Islands | N | Y | N |  |  |
| 6 | ALB | Albania | Y | Y | Y | EURO | Upper middle income |
| 7 | AND | Andorra | Y | Y | Y | EURO | High income |
| 8 | ARE | United Arab Emirates | Y | Y | Y | EMRO | High income |
| 9 | ARG | Argentina | Y | Y | Y | PAHO | Upper middle income |
| 10 | ARM | Armenia | Y | Y | Y | EURO | Upper middle income |
| 11 | ASM | American Samoa | Y | Y | N |  | Upper middle income |
| 12 | ATA | Antarctica | N | Y | N |  |  |
| 13 | ATF | French Southern Territories | N | Y | N |  |  |
| 14 | ATG | Antigua and Barbuda | Y | Y | Y | PAHO | High income |
| 15 | AUS | Australia | Y | Y | Y | WPRO | High income |
| 16 | AUT | Austria | Y | Y | Y | EURO | High income |
| 17 | AZE | Azerbaijan | Y | Y | Y | EURO | Upper middle income |
| 18 | BDI | Burundi | Y | Y | Y | AFRO | Low income |
| 19 | BEL | Belgium | Y | Y | Y | EURO | High income |
| 20 | BEN | Benin | Y | Y | Y | AFRO | Lower middle income |
| 21 | BES_B | Bonaire | Y | Y | N |  |  |
| 22 | BES_E | Sint Eustatius | Y | Y | N |  |  |
| 23 | BES_S | Saba | Y | Y | N |  |  |
| 24 | BFA | Burkina Faso | Y | Y | Y | AFRO | Low income |
| 25 | BGD | Bangladesh | Y | Y | Y | SEARO | Lower middle income |
| 26 | BGR | Bulgaria | Y | Y | Y | EURO | Upper middle income |
| 27 | BHR | Bahrain | Y | Y | Y | EMRO | High income |
| 28 | BHS | Bahamas | Y | Y | Y | PAHO | High income |
| 29 | BIH | Bosnia and Herzegovina | Y | Y | Y | EURO | Upper middle income |
| 30 | BLM | Saint Barthélemy | Y | Y | N |  |  |
| 31 | BLR | Belarus | Y | Y | Y | EURO | Upper middle income |
| 32 | BLZ | Belize | Y | Y | Y | PAHO | Upper middle income |
| 33 | BMU | Bermuda | Y | Y | N |  | High income |
| 34 | BOL | Bolivia, Plurinational State of | Y | Y | Y | PAHO | Lower middle income |
| 35 | BRA | Brazil | Y | Y | Y | PAHO | Upper middle income |
| 36 | BRB | Barbados | Y | Y | Y | PAHO | High income |
| 37 | BRN | Brunei Darussalam | Y | Y | Y | WPRO | High income |
| 38 | BTN | Bhutan | Y | Y | Y | SEARO | Lower middle income |
| 39 | BVT | Bouvet Island | N | Y | N |  |  |
| 40 | BWA | Botswana | Y | Y | Y | AFRO | Upper middle income |
| 41 | CAF | Central African Republic | Y | Y | Y | AFRO | Low income |
| 42 | CAN | Canada | Y | Y | Y | PAHO | High income |
| 43 | CCK | Cocos (Keeling) Islands | N | Y | N |  |  |
| 44 | CHE | Switzerland | Y | Y | Y | EURO | High income |
| 45 | CHL | Chile | Y | Y | Y | PAHO | High income |
| 46 | CHN | China | Y | Y | Y | WPRO | Upper middle income |
| 47 | CIV | Côte d'Ivoire | Y | Y | Y | AFRO | Lower middle income |
| 48 | CMR | Cameroon | Y | Y | Y | AFRO | Lower middle income |
| 49 | COD | Congo, Democratic Republic of the | Y | Y | Y | AFRO | Low income |
| 50 | COG | Congo | Y | Y | Y | AFRO | Lower middle income |
| 51 | COK | Cook Islands | Y | Y | Y | WPRO | High income |
| 52 | COL | Colombia | Y | Y | Y | PAHO | Upper middle income |
| 53 | COM | Comoros | Y | Y | Y | AFRO | Lower middle income |
| 54 | CPV | Cabo Verde | Y | Y | Y | AFRO | Lower middle income |
| 55 | CRI | Costa Rica | Y | Y | Y | PAHO | Upper middle income |
| 56 | CUB | Cuba | Y | Y | Y | PAHO | Upper middle income |
| 57 | CUW | Curaçao | Y | Y | N |  | High income |
| 58 | CXR | Christmas Island | N | Y | N |  |  |
| 59 | CYM | Cayman Islands | Y | Y | N |  | High income |
| 60 | CYP | Cyprus | Y | Y | Y | EURO | High income |
| 61 | CZE | Czechia | Y | Y | Y | EURO | High income |
| 62 | DEU | Germany | Y | Y | Y | EURO | High income |
| 63 | DJI | Djibouti | Y | Y | Y | EMRO | Lower middle income |
| 64 | DMA | Dominica | Y | Y | Y | PAHO | Upper middle income |
| 65 | DNK | Denmark | Y | Y | Y | EURO | High income |
| 66 | DOM | Dominican Republic | Y | Y | Y | PAHO | Upper middle income |
| 67 | DZA | Algeria | Y | Y | Y | AFRO | Lower middle income |
| 68 | ECU | Ecuador | Y | Y | Y | PAHO | Upper middle income |
| 69 | EGY | Egypt | Y | Y | Y | EMRO | Lower middle income |
| 70 | ERI | Eritrea | Y | Y | Y | AFRO | Low income |
| 71 | ESH | Western Sahara | N | Y | N |  |  |
| 72 | ESP | Spain | Y | Y | Y | EURO | High income |
| 73 | EST | Estonia | Y | Y | Y | EURO | High income |
| 74 | ETH | Ethiopia | Y | Y | Y | AFRO | Low income |
| 75 | FIN | Finland | Y | Y | Y | EURO | High income |
| 76 | FJI | Fiji | Y | Y | Y | WPRO | Upper middle income |
| 77 | FLK | Falkland Islands (Malvinas) | Y | Y | N |  |  |
| 78 | FRA | France | Y | Y | Y | EURO | High income |
| 79 | FRO | Faroe Islands | Y | Y | N |  | High income |
| 80 | FSM | Micronesia, Federated States of | Y | Y | Y | WPRO | Lower middle income |
| 81 | GAB | Gabon | Y | Y | Y | AFRO | Upper middle income |
| 82 | GBR | United Kingdom of Great Britain and Northern Ireland | Y | Y | Y | EURO | High income |
| 83 | GEO | Georgia | Y | Y | Y | EURO | Upper middle income |
| 84 | GGY | Guernsey | Y | Y | N |  |  |
| 85 | GHA | Ghana | Y | Y | Y | AFRO | Lower middle income |
| 86 | GIB | Gibraltar | Y | Y | N |  | High income |
| 87 | GIN | Guinea | Y | Y | Y | AFRO | Low income |
| 88 | GLP | Guadeloupe | Y | Y | N |  |  |
| 89 | GMB | Gambia | Y | Y | Y | AFRO | Low income |
| 90 | GNB | Guinea-Bissau | Y | Y | Y | AFRO | Low income |
| 91 | GNQ | Equatorial Guinea | Y | Y | Y | AFRO | Upper middle income |
| 92 | GRC | Greece | Y | Y | Y | EURO | High income |
| 93 | GRD | Grenada | Y | Y | Y | PAHO | Upper middle income |
| 94 | GRL | Greenland | Y | Y | N |  | High income |
| 95 | GTM | Guatemala | Y | Y | Y | PAHO | Upper middle income |
| 96 | GUF | French Guiana | Y | Y | N |  |  |
| 97 | GUM | Guam | Y | Y | N |  | High income |
| 98 | GUY | Guyana | Y | Y | Y | PAHO | Upper middle income |
| 99 | HKG | Hong Kong | Y | Y | N |  | High income |
| 100 | HMD | Heard Island and McDonald Islands | N | Y | N |  |  |
| 101 | HND | Honduras | Y | Y | Y | PAHO | Lower middle income |
| 102 | HRV | Croatia | Y | Y | Y | EURO | High income |
| 103 | HTI | Haiti | Y | Y | Y | PAHO | Low income |
| 104 | HUN | Hungary | Y | Y | Y | EURO | High income |
| 105 | IDN | Indonesia | Y | Y | Y | SEARO | Upper middle income |
| 106 | IMN | Isle of Man | Y | Y | N |  | High income |
| 107 | IND | India | Y | Y | Y | SEARO | Lower middle income |
| 108 | IOT | British Indian Ocean Territory | N | Y | N |  |  |
| 109 | IRL | Ireland | Y | Y | Y | EURO | High income |
| 110 | IRN | Iran, Islamic Republic of | Y | Y | Y | EMRO | Upper middle income |
| 111 | IRQ | Iraq | Y | Y | Y | EMRO | Upper middle income |
| 112 | ISL | Iceland | Y | Y | Y | EURO | High income |
| 113 | ISR | Israel | Y | Y | Y | EURO | High income |
| 114 | ITA | Italy | Y | Y | Y | EURO | High income |
| 115 | JAM | Jamaica | Y | Y | Y | PAHO | Upper middle income |
| 116 | JEY | Jersey | Y | Y | N |  |  |
| 117 | JOR | Jordan | Y | Y | Y | EMRO | Upper middle income |
| 118 | JPN | Japan | Y | Y | Y | WPRO | High income |
| 119 | KAZ | Kazakhstan | Y | Y | Y | EURO | Upper middle income |
| 120 | KEN | Kenya | Y | Y | Y | AFRO | Lower middle income |
| 121 | KGZ | Kyrgyzstan | Y | Y | Y | EURO | Lower middle income |
| 122 | KHM | Cambodia | Y | Y | Y | WPRO | Lower middle income |
| 123 | KIR | Kiribati | Y | Y | Y | WPRO | Lower middle income |
| 124 | KNA | Saint Kitts and Nevis | Y | Y | Y | PAHO | High income |
| 125 | KOR | Korea, Republic of | Y | Y | Y | WPRO | High income |
| 126 | KWT | Kuwait | Y | Y | Y | EMRO | High income |
| 127 | LAO | Lao People's Democratic Republic | Y | Y | Y | WPRO | Lower middle income |
| 128 | LBN | Lebanon | Y | Y | Y | EMRO | Upper middle income |
| 129 | LBR | Liberia | Y | Y | Y | AFRO | Low income |
| 130 | LBY | Libya | Y | Y | Y | EMRO | Upper middle income |
| 131 | LCA | Saint Lucia | Y | Y | Y | PAHO | Upper middle income |
| 132 | LIE | Liechtenstein | Y | Y | N |  | High income |
| 133 | LKA | Sri Lanka | Y | Y | Y | SEARO | Lower middle income |
| 134 | LSO | Lesotho | Y | Y | Y | AFRO | Lower middle income |
| 135 | LTU | Lithuania | Y | Y | Y | EURO | High income |
| 136 | LUX | Luxembourg | Y | Y | Y | EURO | High income |
| 137 | LVA | Latvia | Y | Y | Y | EURO | High income |
| 138 | MAC | Macao | Y | Y | N |  | High income |
| 139 | MAF | Saint Martin (French part) | Y | Y | N |  | High income |
| 140 | MAR | Morocco | Y | Y | Y | EMRO | Lower middle income |
| 141 | MCO | Monaco | Y | Y | Y | EURO | High income |
| 142 | MDA | Moldova, Republic of | Y | Y | Y | EURO | Lower middle income |
| 143 | MDG | Madagascar | Y | Y | Y | AFRO | Low income |
| 144 | MDV | Maldives | Y | Y | Y | SEARO | Upper middle income |
| 145 | MEX | Mexico | Y | Y | Y | PAHO | Upper middle income |
| 146 | MHL | Marshall Islands | Y | Y | Y | WPRO | Upper middle income |
| 147 | MKD | North Macedonia | Y | Y | Y | EURO | Upper middle income |
| 148 | MLI | Mali | Y | Y | Y | AFRO | Low income |
| 149 | MLT | Malta | Y | Y | Y | EURO | High income |
| 150 | MMR | Myanmar | Y | Y | Y | SEARO | Lower middle income |
| 151 | MNE | Montenegro | Y | Y | Y | EURO | Upper middle income |
| 152 | MNG | Mongolia | Y | Y | Y | WPRO | Lower middle income |
| 153 | MNP | Northern Mariana Islands | Y | Y | N |  | High income |
| 154 | MOZ | Mozambique | Y | Y | Y | AFRO | Low income |
| 155 | MRT | Mauritania | Y | Y | Y | AFRO | Lower middle income |
| 156 | MSR | Montserrat | Y | Y | N |  |  |
| 157 | MTQ | Martinique | Y | Y | N |  |  |
| 158 | MUS | Mauritius | Y | Y | Y | AFRO | High income |
| 159 | MWI | Malawi | Y | Y | Y | AFRO | Low income |
| 160 | MYS | Malaysia | Y | Y | Y | WPRO | Upper middle income |
| 161 | MYT | Mayotte | Y | Y | N |  |  |
| 162 | NAM | Namibia | Y | Y | Y | AFRO | Upper middle income |
| 163 | NCL | New Caledonia | Y | Y | N |  | High income |
| 164 | NER | Niger | Y | Y | Y | AFRO | Low income |
| 165 | NFK | Norfolk Island | N | Y | N |  |  |
| 166 | NGA | Nigeria | Y | Y | Y | AFRO | Lower middle income |
| 167 | NIC | Nicaragua | Y | Y | Y | PAHO | Lower middle income |
| 168 | NIU | Niue | Y | Y | Y | WPRO | Upper middle income |
| 169 | NLD | Netherlands, Kingdom of the | Y | Y | Y | EURO | High income |
| 170 | NOR | Norway | Y | Y | Y | EURO | High income |
| 171 | NPL | Nepal | Y | Y | Y | SEARO | Lower middle income |
| 172 | NRU | Nauru | Y | Y | Y | WPRO | High income |
| 173 | NZL | New Zealand | Y | Y | Y | WPRO | High income |
| 174 | OMN | Oman | Y | Y | Y | EMRO | High income |
| 175 | PAK | Pakistan | Y | Y | Y | EMRO | Lower middle income |
| 176 | PAN | Panama | Y | Y | Y | PAHO | High income |
| 177 | PCN | Pitcairn | Y | Y | N |  |  |
| 178 | PER | Peru | Y | Y | Y | PAHO | Upper middle income |
| 179 | PHL | Philippines | Y | Y | Y | WPRO | Lower middle income |
| 180 | PLW | Palau | Y | Y | Y | WPRO | High income |
| 181 | PNG | Papua New Guinea | Y | Y | Y | WPRO | Lower middle income |
| 182 | POL | Poland | Y | Y | Y | EURO | High income |
| 183 | PRI | Puerto Rico | Y | Y | N |  | High income |
| 184 | PRK | Korea, Democratic People's Republic of | Y | Y | Y | SEARO | Low income |
| 185 | PRT | Portugal | Y | Y | Y | EURO | High income |
| 186 | PRY | Paraguay | Y | Y | Y | PAHO | Upper middle income |
| 187 | PSE | Palestine, State of | Y | Y | N |  | Lower middle income |
| 188 | PYF | French Polynesia | Y | Y | N |  | High income |
| 189 | QAT | Qatar | Y | Y | Y | EMRO | High income |
| 190 | REU | Réunion | Y | Y | N |  |  |
| 191 | ROU | Romania | Y | Y | Y | EURO | High income |
| 192 | RUS | Russian Federation | Y | Y | Y | EURO | Upper middle income |
| 193 | RWA | Rwanda | Y | Y | Y | AFRO | Low income |
| 194 | SAU | Saudi Arabia | Y | Y | Y | EMRO | High income |
| 195 | SDN | Sudan | Y | Y | Y | EMRO | Low income |
| 196 | SEN | Senegal | Y | Y | Y | AFRO | Lower middle income |
| 197 | SGP | Singapore | Y | Y | Y | WPRO | High income |
| 198 | SGS | South Georgia and the South Sandwich Islands | N | Y | N |  |  |
| 199 | SHN | Saint Helena, Ascension and Tristan da Cunha | Y | Y | N |  |  |
| 200 | SJM | Svalbard and Jan Mayen | N | Y | N |  |  |
| 201 | SLB | Solomon Islands | Y | Y | Y | WPRO | Lower middle income |
| 202 | SLE | Sierra Leone | Y | Y | Y | AFRO | Low income |
| 203 | SLV | El Salvador | Y | Y | Y | PAHO | Lower middle income |
| 204 | SMR | San Marino | Y | Y | Y | EURO | High income |
| 205 | SOM | Somalia | Y | Y | Y | EMRO | Low income |
| 206 | SPM | Saint Pierre and Miquelon | Y | Y | N |  |  |
| 207 | SRB | Serbia | Y | Y | Y | EURO | Upper middle income |
| 208 | SSD | South Sudan | Y | Y | Y | AFRO | Low income |
| 209 | STP | Sao Tome and Principe | Y | Y | Y | AFRO | Lower middle income |
| 210 | SUR | Suriname | Y | Y | Y | PAHO | Upper middle income |
| 211 | SVK | Slovakia | Y | Y | Y | EURO | High income |
| 212 | SVN | Slovenia | Y | Y | Y | EURO | High income |
| 213 | SWE | Sweden | Y | Y | Y | EURO | High income |
| 214 | SWZ | Eswatini | Y | Y | Y | AFRO | Lower middle income |
| 215 | SXM | Sint Maarten (Dutch part) | Y | Y | N |  | High income |
| 216 | SYC | Seychelles | Y | Y | Y | AFRO | High income |
| 217 | SYR | Syrian Arab Republic | Y | Y | Y | EMRO | Low income |
| 218 | TCA | Turks and Caicos Islands | Y | Y | N |  | High income |
| 219 | TCD | Chad | Y | Y | Y | AFRO | Low income |
| 220 | TGO | Togo | Y | Y | Y | AFRO | Low income |
| 221 | THA | Thailand | Y | Y | Y | SEARO | Upper middle income |
| 222 | TJK | Tajikistan | Y | Y | Y | EURO | Low income |
| 223 | TKL | Tokelau | N | Y | N |  |  |
| 224 | TKM | Turkmenistan | Y | Y | Y | EURO | Upper middle income |
| 225 | TLS | Timor-Leste | Y | Y | Y | SEARO | Lower middle income |
| 226 | TON | Tonga | Y | Y | Y | WPRO | Upper middle income |
| 227 | TTO | Trinidad and Tobago | Y | Y | Y | PAHO | High income |
| 228 | TUN | Tunisia | Y | Y | Y | EMRO | Lower middle income |
| 229 | TUR | Türkiye | Y | Y | Y | EURO | Upper middle income |
| 230 | TUV | Tuvalu | Y | Y | Y | WPRO | Upper middle income |
| 231 | TWN | Taiwan, Province of China | Y | Y | N |  |  |
| 232 | TZA | Tanzania, United Republic of | Y | Y | Y | AFRO | Lower middle income |
| 233 | UGA | Uganda | Y | Y | Y | AFRO | Low income |
| 234 | UKR | Ukraine | Y | Y | Y | EURO | Lower middle income |
| 235 | UMI | United States Minor Outlying Islands | N | Y | N |  |  |
| 236 | URY | Uruguay | Y | Y | Y | PAHO | High income |
| 237 | USA | United States of America | Y | Y | Y | PAHO | High income |
| 238 | UZB | Uzbekistan | Y | Y | Y | EURO | Lower middle income |
| 239 | VAT | Holy See | N | Y | N |  |  |
| 240 | VCT | Saint Vincent and the Grenadines | Y | Y | Y | PAHO | Upper middle income |
| 241 | VEN | Venezuela, Bolivarian Republic of | Y | Y | Y | PAHO | Upper middle income |
| 242 | VGB | Virgin Islands (British) | Y | Y | N |  | High income |
| 243 | VIR | Virgin Islands (U.S.) | Y | Y | N |  | High income |
| 244 | VNM | Viet Nam | Y | Y | Y | WPRO | Lower middle income |
| 245 | VUT | Vanuatu | Y | Y | Y | WPRO | Lower middle income |
| 246 | WLF | Wallis and Futuna | Y | Y | N |  |  |
| 247 | WSM | Samoa | Y | Y | Y | WPRO | Upper middle income |
| 248 | XKX | Kosovo | Y | Y | N |  | Upper middle income |
| 249 | YEM | Yemen | Y | Y | Y | EMRO | Low income |
| 250 | ZAF | South Africa | Y | Y | Y | AFRO | Upper middle income |
| 251 | ZMB | Zambia | Y | Y | Y | AFRO | Lower middle income |
| 252 | ZWE | Zimbabwe | Y | Y | Y | AFRO | Lower middle income |

The CTA targeted by each measure was mapped from the fields TARGET_COUNTRY, TARGET_REGION, TARGET_CITY, TARGET_PROVINCE, and TARGET_OTHER_GEO. Each of the 250 target CTAs were given a Boolean variable to indicate if they were targeted by a given measure (1) or not (0). A target all Boolean indicated when the target description read “All countries”. CTAs were not able to self-target and 197 measures were unmapped due to exclusive self-targeting.

Most measures were mapped to all CTAs or by using ISOs in these fields. A minority of measures targeted a collection of Member States based on participation in regional organizations. Table 7 describes how the associated targeted CTAs were mapped. In addition to these, about 100 measures defined targets based on macro regions such as Asia, East Asia, Africa, Europe, Central America, and North America. Given these accounted for only 1% of all measures and the disputed borders of these regions, we opted not to map them to CTAs.

*Table 7*

| **European Union** | **Non-European Union** |
| --- | --- |
| Austria, Belgium, Bulgaria, Croatia, Cyprus, Czechia, Denmark, Estonia, Finland, France, Germany, Greece, Hungary, Italy, Ireland, Latvia, Lithuania, Luxembourg, Malta, Netherlands, Poland, Portugal, Slovakia, Slovenia, Romania, Spain, Sweden | All other CTAs |
|  |  |
| **European Economic Area** | **Non-European Economic Area** |
| Austria, Belgium, Bulgaria, Croatia, Cyprus, Czechia, Denmark, Estonia, Finland, France, Germany, Greece, Hungary, Iceland, Italy, Ireland, Latvia, Liechtenstein, Lithuania, Luxembourg, Malta, Netherlands, Poland, Portugal, Slovakia, Slovenia, Romania, Spain, Sweden | All other CTAs |
|  |  |
| **Schengen** | **Non-Schengen** |
| Austria, Belgium, Czechia, Denmark, Estonia, Finland, France, Germany, Greece, Hungary, Iceland, Italy, Latvia, Liechtenstein, Lithuania, Luxembourg, Malta, Netherlands, Poland, Portugal, Slovakia, Slovenia, Spain, Sweden, Switzerland | All other CTAs |
|  |  |
| **Third Countries** |  |
| Non-EU and non-Schengen countries |  |
|  |  |
| **Gulf Cooperation Council** | **Non-GCC** |
| Bahrain, Kuwait, Oman, Qatar, Saudi Arabia, United Arab Emirates | All other CTAs |
|  |  |
| **AESEAN** |  |
| Brunei Darussalam, Cambodia, Indonesia, Lao People's Democratic Republic, Malaysia, Myanmar, Philippines, Singapore, Thailand, Viet Nam |  |

When measures targeted, with exceptions, all CTAs or all members/non-member of a supranational organization, then all Booleans in the group would be set to 1 and non-participants and exceptions would be set to 0. The target all Boolean would also be set to 0. For instance, “Non-EU and Non-Schengen except GBR” would have all Non-EU and Non-Schengen CTAs set to 1, and the EU, Schengen, GBR, and target all Boolean set to 0.

Where possible, if a measure targeted a sub-region not included in the ISO-3 list, it was mapped to its associated CTA. For instance, Wuhan was mapped to China. Mapping from city to CTA was done using the Basic World Cities Database version 1.76 (Pareto Software, n.d.). Where cities of the same name exist in multiple countries (e.g., London), the most populous city was chosen and/or informed by information in the original PHSM entry. Additional cities and regions not in the above database are shown in Table XX. This mapping was generated by an RA using an AI tool with manual checks.

*Table 8: Mapping of additional cities and regions not in World Cities Database*

| **Region** | **Mapped ISO** | **Region** | **Mapped ISO** |
| --- | --- | --- | --- |
| Abrruzo | ITA | Mazandaran | IRN |
| Achaea | GRC | Mazandaran Province | IRN |
| Addis Abab | ETH | Međimurje | HRV |
| Agder | NOR | Medimurska | HRV |
| Alabama | USA | Mehedinti | ROU |
| Aland | FIN | Menorca | ESP |
| Alaska | USA | Michigan | USA |
| Alba | ITA | Mid-East |  |
| Alborz | IRN | Miglia |  |
| Alborz Province | IRN | Minnesota | USA |
| Algeris | DZA | Mira |  |
| all Finnish regions with the exception of Etela-Saavo | FIN | Mississippi | USA |
| Alpes |  | Missouri | USA |
| Alpes-Maritimes | FRA | Mittelberg/Kleinwalsertal | AUT |
| and Baghdad | IRQ | Montana | USA |
| and Cote d'Azur | FRA | Møre og Romsdal | NOR |
| and Zhejiang | CHN | Møre og Romsdal and Nord-Trøndelag | NOR |
| Andong | KOR | Moselle | FRA |
| Andong-si | KOR | Mugla | TUR |
| Antalya | TUR | Mykonos | GRC |
| Antwerp | BEL | Nairobi | KEN |
| Aosta Valley | ITA | Nakhchivan | AZE |
| Apulia | ITA | Nangarhar | AFG |
| Aragon | ESP | Naples | ITA |
| Arges | ROU | Navarra | ESP |
| Ariège and Pyrnénées-Orientales | FRA | Navarre | ESP |
| Arizona | USA | Neamt | ROU |
| Arkansas | USA | Nebraska | USA |
| Athens | GRC | Neuchatel | CHE |
| Atlanta | USA | Nevada | USA |
| Attica | GRC | New Aquitaine | FRA |
| Auvergne-Rhone-Alpes | FRA | New Hampshire | USA |
| Aydin | TUR | New Jersey | USA |
| Azores Islands | PRT | New Mexico | USA |
| Bacau | ROU | New South Wales | AUS |
| Balearic and Canary Islands | ESP | New York | USA |
| Balearic Islands | ESP | Nidwalden | CHE |
| Baranya | HUN | Niederosterreich | AUT |
| Basel-Stadt. | CHE | Nitra | SVK |
| Basilicata | ITA | Nograd | HUN |
| Basque | ESP | Nógrád | HUN |
| Basque Country | ESP | Noord-Holland | NLD |
| Belgian | BEL | Nordjylland | DNK |
| Belladere | DOM | Nordland | NOR |
| Berlin | DEU | Normandie | FRA |
| Bertonico | ITA | Normandy | FRA |
| Bethlehem | PSE | Norrbotten | SWE |
| Bihor | ROU | Norte | PRT |
| Bjelovar-Bilogora | HRV | North Aegean | GRC |
| Blagoevgrad | BGR | North Carolina | USA |
| Blekinge | SWE | North Dakota | USA |
| Blekinge | SWE | North Gyeongsang | KOR |
| Bolzano – South Tyrol | ITA | North Gyeongsang Province | KOR |
| Borsod-Abauj-Zemplen | HUN | North Holland | NLD |
| Borsod-Abaúj-Zemplén | HUN | North Karelia | FIN |
| Bouches-du-Rhone | FRA | North Ostrobothnia | FIN |
| Braila | ROU | Northen Italy | ITA |
| Brandenburg | DEU | Northern Iran | IRN |
| Brasov | ROU | Northern Ireland | GBR |
| Bratislava | SVK | northern ITA | ITA |
| Bretagne | FRA | Northern Italy | ITA |
| British Overseas Territory | GBR | Northern Ostrobothnia | FIN |
| Brittany | FRA | Northern Savolax | FIN |
| Brodsko-Posavska | HRV | Northern Savonia | FIN |
| Budapest | HUN | Nouvelle-Aquitaine | FRA |
| Burgenland | AUT | Oberosterreich | AUT |
| Buzau | ROU | Occitane | FRA |
| California | USA | Occitanie | FRA |
| Campania | ITA | Ohio | USA |
| Canary Islands | ESP | Oklahoma | USA |
| Canton of Ticino | CHE | Opole | POL |
| Caras Sever | ROU | Orebro | SWE |
| Casalpusterlengo | ITA | Örebro | SWE |
| Castelgerundo | ITA | Oregon | USA |
| Castiglione d'Adda | ESP | Osijek-Baranja | HRV |
| Catalonia | ESP | Oslo | NOR |
| Center-Val de Loire | FRA | Oslo | NOR |
| Central Finland | FIN | Osrednjeslovenska | SVN |
| Central Greece | GRC | Ostergotland | SWE |
| Central Hämeenlinna | FIN | Östergötland | SWE |
| Central Jutland | DNK | Ostrobothnia | FIN |
| Central Macedonia | GRC | Ostrobothnia and other risk areas | FIN |
| Central Ostrobothnia and Southern Ostrobothnia | FIN | Ouanaminthe | DOM |
| Centre-Val de Loire | FRA | P lva |  |
| Centro | ITA | Päijänne-Häme (Päijät Häme) | FIN |
| Chaman | PAK | Päijänne-Häme Päijät Häme) | FIN |
| Chaman | PAK | Päijät-Häme | FIN |
| chandigarh | IND | Paris | FRA |
| Channel Island of Jersey | GBR | parts of Croatia | HRV |
| Cheondo-gun | KOR | Pays de la Loire | FRA |
| Cheongdo | KOR | Peloponnese | GRC |
| Chilgok | KOR | Pennsylvania | USA |
| Chilgok-gun | KOR | Pesaro-Urbino | ITA |
| Cities in the USA that have documneted community spread | USA | Pest | HUN |
| Clujm | ROU | Piedmont | ITA |
| Codogno | ITA | Piemonte | ITA |
| Colorado | USA | Piemonte Region | ITA |
| Connecticut | USA | Pirkanmaa | FIN |
| Corse | FRA | Podkarpackie | POL |
| Corsica | FRA | Podlaski | POL |
| Covasna | ROU | Podravska | SVN |
| Crete | GRC | Pohjanmaa | FIN |
| Crimea |  | Pohjois-Savo | FIN |
| Crystal City | USA | Pomorskie | POL |
| Csongrad-Csanad | HUN | Pomurska | SVN |
| Csongrád-Csanád | HUN | Porto | PRT |
| Daegu | KOR | Posavska | HRV |
| Daegu city | CHN | Pozega-Slavonia | HRV |
| Daegu-guangyeok-si | KOR | Prague | CZE |
| Dahka | BGD | Prahova | ROU |
| Dalarna | SWE | Presov | SVK |
| Dambovita | ROU | Primorsko-notranjska | SVN |
| District of Columbia | USA | Provence | FRA |
| Dobritch | BGR | Provence-Alpes-Cote d'Azur | FRA |
| Dobritsch | BGR | Puerto Falcón | PRY |
| Dortmund | DEU | Qazvin Province | IRN |
| Dublin | IRL | Qom | IRN |
| Dubrovnik-Neretva | HRV | Qom Province | IRN |
| East Lancashire | GBR | Rapla | EST |
| East Midlands | GBR | Rasgrad | BGR |
| Eastern Macedonia and Thrace | GRC | Red and orange zones in ITA | ITA |
| Elis | GRC | Rhode Island | USA |
| Emilia Romagna | ITA | Riga | LVA |
| Emilia Romagna Region | ITA | Rimini | ITA |
| Emilia-Romagna | ITA | Rogaland | NOR |
| England | GBR | Romagna | ITA |
| England | GBR | Sachsen | DEU |
| Epirus | GRC | Saint-Barthélemy | FRA |
| Etela Savo | FIN | Salzburg | AUT |
| Etela-Karjala | FIN | San Fiorano | ITA |
| Finland: hospital district of Kajanaland | FIN | Santorini | GRC |
| Finland: hospital district of Kainuu | FIN | Sardinia | ITA |
| Finland: hospital district of Länsi-Pohja | FIN | Sardinien | ITA |
| Finland: hospital district of Norra Savolax | FIN | Sarthe | FRA |
| Finland: hospital district of North Karelia | FIN | Satakunta | FIN |
| Finland: hospital district of South Karelia | FIN | Savinjska | SVN |
| Finland: hospital districts of Central Häme | FIN | Saxony | DEU |
| Finland: hospital districts of Central Ostrobothnia | FIN | Saxony-Anhalt | DEU |
| Finland: hospital districts of Central Ostrobothnia Hospital and the North Karelia | FIN | Scania | SWE |
| Finland: hospital districts of Etelä-Savo nd Pohjois-Savo | FIN | Schleswig-Holstein | DEU |
| Finland: hospital districts of Länsi-Pohja | FIN | Schwyz | CHE |
| Finland: South Ostrobothnia | FIN | Scotland | GBR |
| Finland: the hospital district of Lapland | FIN | Seine-Saint-Denis | FRA |
| Finland: the hospital districts of Central Finland | FIN | Semnan | IRN |
| Finland: the hospital districts of Central Ostrobothnia | FIN | Semnan Province | IRN |
| Finland: Uusimaa Uusimaa) | FIN | Seongju | KOR |
| Florence | ITA | Seongju-gun | KOR |
| Floria | USA | Serifos | GRC |
| Florida | USA | Siauliu | LTU |
| Fombio | ITA | Šiaulių | LTU |
| Foz do Iguazu | BRA | Sibensko-kninska | HRV |
| Freiburg | DEU | Sichuan | CHN |
| Fribourg | CHE | Sicily | ITA |
| Friuli Venezia Giulia | ITA | Silesian Voivodeship | POL |
| Friuli-Venezia Giulia | ITA | Sisacko-moslavacka | HRV |
| Galati | ROU | Sisak-Moslavina | HRV |
| Gaza strip | PSE | Skane | SWE |
| Gazvin | IRN | Skåne | SWE |
| Geelan | IRN | Sliven | BGR |
| Geneva | CHE | Södermanland | SWE |
| Georgia | USA | Sofia | BGR |
| Gilan | IRN | Sofia City | BGR |
| Gilan Province | IRN | Somaglia | ITA |
| Girona | KOR | Somogy | HUN |
| Golestan | IRN | Sörmland | SWE |
| Golestan Province | IRN | South Carolina | USA |
| Gorenjska | SVN | South Dakota | USA |
| Goriska | SVN | South Holland | NLD |
| Gorj | ROU | South Karelia and South Ostrobothnia | FIN |
| Grad Zagreb | HRV | South Ossetia |  |
| Grampian Territorial Health District | GBR | South Ostrobothnia | FIN |
| Greater Manchester | GBR | South-East region in Ireland | IRL |
| Guadeloupe | FRA | Southern Ostrobothnia | FIN |
| Guangdong | CHN | Southern Savonia | FIN |
| Gunwi | KOR | South-West |  |
| Gunwi-gun in Gyeongsangbuk-do | KOR | Southwest Finland | FIN |
| Gütersloh district | DEU | Split-Dalmatia | HRV |
| Guyane | FRA | Split-Dalmatia County | HRV |
| Gyeongbuk | KOR | St. Petersburg | RUS |
| Gyeongsan | KOR | Steiermark | AUT |
| Gyeongsan City | ESP | Stockholm | SWE |
| Gyeongsangbuk-do | KOR | Stredocesky | CZE |
| Gyeongsan-si | KOR | Surrounding ares of Wuhan | CHN |
| Gyor-Moson-Sopron | HUN | Sweden: Blekinge and Södermanland | SWE |
| Győr-Moson-Sopron | HUN | Sweden: Blekinge and Södermanland; Finland: Uusimaa (Uusimaa) | SWE |
| Hajdu-Bihar | HUN | Sweden: Kalmar | SWE |
| Hajdú-Bihar | HUN | Sweden: Östergötland | SWE |
| Halland | SWE | Swietokrzyskie | POL |
| Hamburg | DEU | Szabolcs-Szatmar-Bereg | HUN |
| Harju | EST | Szabolcs-Szatmár-Bereg | HUN |
| Hauts-de-France | FRA | Targovishte | BGR |
| Hawaii | USA | Tartu | EST |
| Hebei | CHN | Teheran | IRN |
| Heilongjiang | CHN | Tehran | IRN |
| Helsiki | FIN | Tehran Province | IRN |
| Helsinki | FIN | Telsiai | LTU |
| Helsinki-Uusimaa and Paijat-Hame | FIN | Tennessee | USA |
| Hérault | FRA | Terranova dei Passerini | ITA |
| Heves | HUN | Texas | USA |
| Hiiu | EST | the Midlands |  |
| Hovedstaden | DNK | The state of Tyrol in Austria | AUT |
| Hubei | CHN | Thuringen | DEU |
| Hubei Province | CHN | Thüringen | DEU |
| Ialomita | ROU | Thuringia | DEU |
| Iasi | ROU | Ticino | CHE |
| Ibenik-Knin | HRV | Timis | ROU |
| Ibiza and Formentera | ESP | Tinos | GRC |
| Idaho | USA | Tirol | AUT |
| Ida-Viru | EST | Tonghua | CHN |
| Ile-de-France | FRA | Torres Strait Regions | AUS |
| Île-de-France | FRA | Toskana | ITA |
| Ilfov | ROU | Trentino-Alto Adige | ITA |
| Inner Mongolia | CHN | Trnava | SVK |
| Innlandet | NOR | Troms and Finnmark | NOR |
| International Peace Garden |  | Trøndelag | NOR |
| Iowa | USA | Tuscany | ITA |
| Ireland | IRL | Tyrol | AUT |
| Ireland Midlands | IRL | Uiseong | KOR |
| Ischgl | AUT | Uiseong-gun | KOR |
| Ischgl | AUT | Umbria | ITA |
| Isfahan | IRN | Umbria | ITA |
| Isfahan Province | IRN | Upper Austria | AUT |
| Islambad | PAK | Uppsala | SWE |
| Islands in the Indian Ocean region |  | Uri | CHE |
| Itä-Savo | FIN | Urumqi | CHN |
| Izmir | TUR | Utah | USA |
| Jamtland | SWE | Utrecht | NLD |
| Jämtland | SWE | Uusimaa | FIN |
| Jasz-Nagykun-Szolnok | HUN | Val-de-Marne | FRA |
| Jász-Nagykun-Szolnok | HUN | Valga | EST |
| Jiangsu | CHN | Valka | LVA |
| Jogeva | EST | Valle d'Aosta | ITA |
| Jonkoping | SWE | Vama | ROU |
| Jönköping | SWE | Varazdin | HRV |
| Jugovzhodna Slovenija | SVN | Varaždin | HRV |
| Jungholz | AUT | Varna | BGR |
| Jura |  | Vas | HUN |
| Kainuu | FIN | Vaslui | ROU |
| Kalmar and Värmland | SWE | Vasterbotten | SWE |
| Kansas | USA | Västerbotten | SWE |
| Kanta-Hame | FIN | Vasternorrland | SWE |
| Karlovac | HRV | Vastmanland and Vastra Gotaland | SWE |
| Katowice | POL | Vaud | CHE |
| Kaunas | LTU | Venetia | ITA |
| Kaunas administrative unit | LTU | Veneto | ITA |
| Kentucky | USA | Veneto Region | ITA |
| Kesice | SVK | Venice | ITA |
| Keski-Pohjanmaa | FIN | Vermont | USA |
| Klaipeda | LTU | Vestland | NOR |
| Komarom-Esztergom | HUN | Veszprem | HUN |
| Komárom-Esztergom | HUN | Veszprém | HUN |
| Koroska | SVN | Vicea | ROU |
| Kraj Zilina | SVK | Victoria | AUS |
| Krakow | POL | Vidzeme | LVA |
| Krapina-Zagorje | HRV | Vienna | AUT |
| Krapinsko-zagorska zupanija | HRV | Viken | NOR |
| Kronoberg | SWE | Viljandi | EST |
| Kujawsko-Pomorskie | POL | Vilnius | LTU |
| La Reunion | FRA | Vilnius | LTU |
| La Rioja | ESP | Virginia | USA |
| Lake Constance |  | Virovitica-Podravina | HRV |
| Länsi-Pohja | FIN | Viroviticko-Podravska | HRV |
| Lapland |  | Vo’ Euganeo | ITA |
| Lappi |  | Vorarlberg | AUT |
| Latgale | LVA | Vrancea | ROU |
| Lazio | ITA | Vru |  |
| le de France | FRA | Vukovarsko-srijemska | HRV |
| Lesser Poland Voivodeship | POL | Vukovar-Srijem County | HRV |
| Lesvos | GRC | Vukovar-Syrmia | HRV |
| Liaoning | CHN | Wales | GBR |
| Liguria | ITA | Washington | USA |
| Lika-Senj | HRV | Washington D.C. | USA |
| Lisbon | PRT | Washington State | USA |
| Lolestan | IRN | West and Midlands |  |
| Lombardi | ITA | West Bank | PSE |
| Lombardia | ITA | West Gothia | SWE |
| Lombardy | ITA | West Midlands | GBR |
| Lombardy and Veneto | ITA | West of Ireland | IRL |
| Lombardy Region | ITA | West Yorkshire | GBR |
| London Heathrow | GRB | Western Balkan |  |
| Lorestan Province | ITA | western Greece | GRC |
| Louisiana | USA | Western Macedonia | GRC |
| Lower Austria | AUT | Western Macedonia and Thessaly | GRC |
| Lubusz | POL | Wisconsin | USA |
| Madeira | PRT | Wuhan and its surrounding areas | CHN |
| Madrid | ESP | Wyoming | USA |
| Maine | USA | Xi'an | CHN |
| Mainland Spain | ESP | XWB | PSE |
| Maleo | IDN | Yeongcheon | KOR |
| Mallorca | ESP | Yeongcheon-si | KOR |
| Małopolska | POL | Yorkshire and the Humber | GBR |
| Malopolskie | POL | Zadar | HRV |
| Malpasse | DOM | Zadarska | HRV |
| Marche | ITA | Zagreb | HRV |
| Marche Region | ITA | Zakynthos | GRC |
| Marijanpole | UKR | Zala | HUN |
| Markazi Province | IRN | Zasavska | SVN |
| Marqazi | IRN | Zemgale | LVA |
| Martinique | FRA | Zheijang | CHN |
| Maryland | USA | Zug | CHE |
| Massachusetts | USA | Zuid-Holland | NLD |
| Mayotte | FRA | Zurich | CHE |

As detailed in Table 8, we estimate that about 8.4% of our database did not map to any target CTA. About half of these measures, 4.5% of all measures, had descriptions of targets based on a risk level assessment that was not easily discernible from the PHSM dataset, link, and/or additional searches. 1.7% had the same implementing CTA and target CTA (self-targeting), 1.2% did not target a WHO member state. We also identified an additional 1.2% of measures that were partially unmapped, being that some but not all target countries could be identified from the target descriptions. Fewer than 100 measures were partially or fully unmapped because of vague or disputed geographical definitions such as neighboring countries, Europe, or Asia.

After mapping target Booleans, we dropped duplicate records based on implementing CTA, measure type, measure subtype, week of implementation, measure type, measure subtype, and target Booleans. As a hard division between weeks is arbitrary and to avoid having identical records in different weeks, we also dropped duplicate records based on the above variables and with a shift of the start day of the week by three days. Between duplicates, we kept the record with the earliest date start and the original PHSM record over a record added by our coders, if applicable.

Income level was defined using World Bank databases via the Stata package wbopendata, version 16.3 (Azevedo, 2011). Two Member States, Niue and Cook Islands, are not members of the World Bank. They were defined as upper middle income and high income, respectively.

## Limitations and usage notes

Due to the time constraint and the inherent limitations of our data coding approach, the dataset should be used with reservations. Our database was built on an existing dataset whose limitations are previously noted. Given the volume of the data, we did not conduct a systematic search on sources and validate every entry in the PHSM dataset. As a result, our database at best offers a partial view of international travel measures implemented by CTAs, especially for smaller CTAs and CTAs less frequently covered by media outlets. Moreover, some of the issues embedded in PHSM (e.g., errors in the start date of a measure) may still be found in our database. Also, we did not include end date and compliance level as balancing research goals and available resources necessitated putting these variables aside.

Despite the substantial efforts to improve data quality and coding procedures, inconsistencies in data coding are unavoidable. This issue was prominent for measure status, which we ultimately decided not to include measure status in our analyses. That being said, figure 3 excluding measure status “extending” and “finishing” is included in this appendix.

There were also inconsistencies in the judgment on the measure type, particularly for “Closing borders”, “Individual-based travel restriction/ban”, and “CTA-based travel restriction/ban”. The problem originated from three main causes: (1) inconsistent definition and usage of terms; (2) the prominence of non-government source in PHSM (e.g., Garda World), or the website of foreign CTA’s government institution (e.g., embassy) rather than the implementing CTA, resulting in incomplete information; and (3) the majority of international travel measures had exemptions which may have led to a different interpretation on its measure type and/or subtype.

We also observed inconsistencies in measure description and links with the defined target country, region, province, city, and other geography. This was a particular issue for measures whose targets were defined using descriptives, rather than a list of ISOs or were targeting all CTAs. Based on data verification of a random sample of measures, we estimate that this issue impacts less than 3% of records. We fixed any inconsistencies that we found, but ultimately deemed that they had little impact on our analysis.

## Data & Code Availability

The data are available from the corresponding author upon reasonable request.

## 5. Additional Tables and Figures

**Table 9: Summary of measures not mapped to a target Member State**

|  | **(1)** | **(2)** | **(3)** | **(4)** | **(5)** | **(6)** |  |
| --- | --- | --- | --- | --- | --- | --- | --- |
|  | | Measures fully unmapped | % of fully unmapped measures | % of measures | Measures partially unmapped | % partially unmapped measures | % of measures |
| Reason not mapped | |  |  |  |  |  |  |
| Based on risk level | | 531 | 53.3 | 4.6 | 154 | 29.5 | 1.3 |
| Self-targeted | | 191 | 19.2 | 1.7 | 1 | 0.2 | 0.0 |
| Unclear list of countries | | 79 | 7.9 | 0.7 | 285 | 54.6 | 2.5 |
| No defined target | | 76 | 7.6 | 0.7 | 0 | 0 | 0 |
| Unclear regional descriptor | | 63 | 6.3 | 0.6 | 63 | 12.1 | 0.6 |
| Neighbouring countries | | 16 | 1.6 | 0.1 | 19 | 3.6 | 0.2 |
| Other | | 41 | 4.1 | 0.4 | 0 | 0 | 0 |
|  | |  |  |  |  |  |  |
| Total | | 997 | 100 | 8.7 | 522 | 100 | 4.6 |
|  | |  |  |  |  |  |  |

Fully unmapped measures have no definable Member State targets. Partially unmapped measures have at least one definable Member State target but also targeted CTAs that were unmapped.
Unmapped measures were identified using a keyword search on target descriptions, i.e. target country, target region, target population. In a minority of cases, a measure could have been unmapped for multiple reasons. Where there was overlap, priority was first given to unclear regional descriptors and then, in descending order, self-targeted, neighboring countries, measures based on risk levels, and unclear list of countries. Other includes measures that do not have a target region but do not fall into one of the above-described categories.
Measures based on a risk level used the keywords risk, variant, strain, emerging, case, affected, pandemic, endemic, epidemic, epicenter, outbreak, incidence, high level, transmission, infected, safe, red, orange, yellow, green, grey, spread, per 10, symptomatic, temperature, asymptomatic, covid excluding covid level or covid test.
No defined target used the keyword no target. It also includes measures with NA or blanks for all target descriptions.
Measures based on an unclear list of countries used the keywords list, level, no exceptions, negative testing, negative covid-19 test, negative results, avoidance of non-essential travel, over 8h flight away, category, categories, reciprocity, restriction, visa, countries from, countries for, countries to, bubble, ban, designated states, open, quarantine, group, most, multiple, non-corridor, some, Portuguese, Dutch, non-open, [0-9]+ countries. To avoid double counting, it excluded measures already grouped in risk levels
Neighboring countries used the keywords neighboring, neighboring, bordering, countries of the region, outside of the six, sea connections, designated check points, checkpoints
Unclear regional descriptor used keywords Africa, Middle East, East Asia, Southeast Asia, Central Asia, West Asia, Asia, Latin America, North America, South America, Central America, Americas, Caribbean, Gulf countries, Pacific Island countries, Europe, Baltic including non-Baltic, and Nordic including non-Nordic.

**Table 10: Summary of measures, by Member State income levels**

|  | **(1)** | **(2)** | **(3)** | **(4)** | **(5)** | **(6)** | **(7)** | **(8)** |  |
| --- | --- | --- | --- | --- | --- | --- | --- | --- | --- |
|  | | High Income | % of Total | Upper Middle Income | % of Total | Lower Middle Income | % of Total | Lower income | % of Total |
|  | n | % | n | % | n | % | n | % |  |
|  | |  |  |  |  |  |  |  |  |
| **Total number of measures** | | 4772 |  | 2754 |  | 1964 |  | 921 |  |
|  | |  |  |  |  |  |  |  |  |
| **Measure implementation date** | |  |  |  |  |  |  |  |  |
| Earliest date | | 03jan2020 |  | 01jan2020 |  | 02jan2020 |  | 02jan2020 |  |
| Mean date | | 15jul2020 |  | 21jun2020 |  | 20jun2020 |  | 25jun2020 |  |
| Latest date | | 31dec2020 |  | 31dec2020 |  | 30dec2020 |  | 31dec2020 |  |
|  | |  |  |  |  |  |  |  |  |
| **Measure type** | |  |  |  |  |  |  |  |  |
| Quarantine | | 1135 | 23.8 | 497 | 18.0 | 361 | 18.4 | 192 | 20.8 |
| Testing | | 878 | 18.4 | 431 | 15.7 | 315 | 16.0 | 188 | 20.4 |
| Individual-based restriction | | 739 | 15.5 | 379 | 13.8 | 251 | 12.8 | 61 | 6.6 |
| Restricting flights | | 333 | 7.0 | 442 | 16.0 | 297 | 15.1 | 122 | 13.2 |
| Health screening | | 193 | 4.0 | 217 | 7.9 | 175 | 8.9 | 128 | 13.9 |
| CTA-based restriction | | 332 | 7.0 | 144 | 5.2 | 55 | 2.8 | 21 | 2.3 |
| Additional travel documents | | 230 | 4.8 | 153 | 5.6 | 115 | 5.9 | 30 | 3.3 |
| Travel advice or warning | | 350 | 7.3 | 63 | 2.3 | 44 | 2.2 | 24 | 2.6 |
| Border closure | | 66 | 1.4 | 128 | 4.6 | 95 | 4.8 | 77 | 8.4 |
| Other travel restrictions | | 166 | 3.5 | 79 | 2.9 | 59 | 3.0 | 12 | 1.3 |
| Visa-related restriction | | 100 | 2.1 | 72 | 2.6 | 99 | 5.0 | 16 | 1.7 |
| Other types | | 250 | 5.2 | 149 | 5.4 | 98 | 5.0 | 50 | 5.4 |
| **Total** | | 4772 | 100 | 2754 | 100 | 1964 | 100 | 921 | 100 |
|  | |  |  |  |  |  |  |  |  |

Notes: Includes measures implemented by WHO Member States from December 31 2019 to December 31 2020.

**Table 11: Summary of Member States targeting any Member State and targeting all CTAs**

|  | **(1)** | **(2)** | **(3)** | **(4)** |  |
| --- | --- | --- | --- | --- | --- |
| **Category** | Targeting any Member State | % of Member States | Targeting All CTAs | % of Member States |  |
|  | n | % | n | % |  |
| Member states | | 194 | 100 | 194 | 100 |
|  | |  |  |  |  |
| **Measure type** | |  |  |  |  |
| Quarantine | | 192 | 99.0 | 181 | 93.3 |
| Testing | | 182 | 93.8 | 171 | 88.1 |
| Health screening | | 181 | 93.3 | 167 | 86.1 |
| Restricting flights | | 181 | 93.3 | 157 | 80.9 |
| Individual-based restriction | | 175 | 90.2 | 158 | 81.4 |
| Additional travel documents | | 143 | 73.7 | 136 | 70.1 |
| Travel advice or warning | | 115 | 59.3 | 72 | 37.1 |
| Other travel restrictions | | 114 | 58.8 | 88 | 45.4 |
| CTA-based restriction | | 113 | 58.2 | 10 | 5.2 |
| Border closure | | 107 | 55.2 | 101 | 52.1 |
| Visa-related restriction | | 82 | 42.3 | 73 | 37.6 |
| Other types | | 140 | 72.2 | 93 | 47.9 |
|  | |  |  |  |  |

Includes measures from December 31, 2019, to December 31, 2020.

**Table 12: Summary of measure status**

|  | **(1)** | **(2)** | **(3)** | **(4)** | **(5)** | **(6)** |  |
| --- | --- | --- | --- | --- | --- | --- | --- |
|  | | Full year |  | Full year |  | Half year |  |
| **Category** | All | % of Total | Earliest | % of Total | All | % of Total |  |
|  | n | % | n | % | n | % |  |
|  | |  |  |  |  |  |  |
| **Measure status** | |  |  |  |  |  |  |
| Extending | | 3073 | 26.9 | 118 | 5.5 | 1167 | 20.5 |
| Introducing | | 2797 | 24.5 | 1665 | 76.9 | 2095 | 36.8 |
| Strengthening | | 2407 | 21.1 | 178 | 8.2 | 1273 | 22.4 |
| Easing | | 1449 | 12.7 | 77 | 3.6 | 501 | 8.8 |
| Modifying | | 1278 | 11.2 | 103 | 4.8 | 528 | 9.3 |
| Finishing | | 316 | 2.8 | 20 | 0.9 | 114 | 2.0 |
| Re-Introducing | | 111 | 1.0 | 3 | 0.1 | 15 | 0.3 |
| **Total** | | 11431 | 100 | 2164 | 100 | 5693 | 100.0 |
|  | |  |  |  |  |  |  |

Notes: Columns 1 and 5 describe all measures while column 3 describes the first observed measure for each implementing country and measure type [i.e. the first time Australia adopted quarantine]. Columns 1 and 3 cover measures implemented from December 31, 2019, to December 31 2020. Column 5 covers measures implemented from December 31, 2019, to June 30 2020.

Figure 2: Number of Member States adopting measures, by week of adoption, full year of data


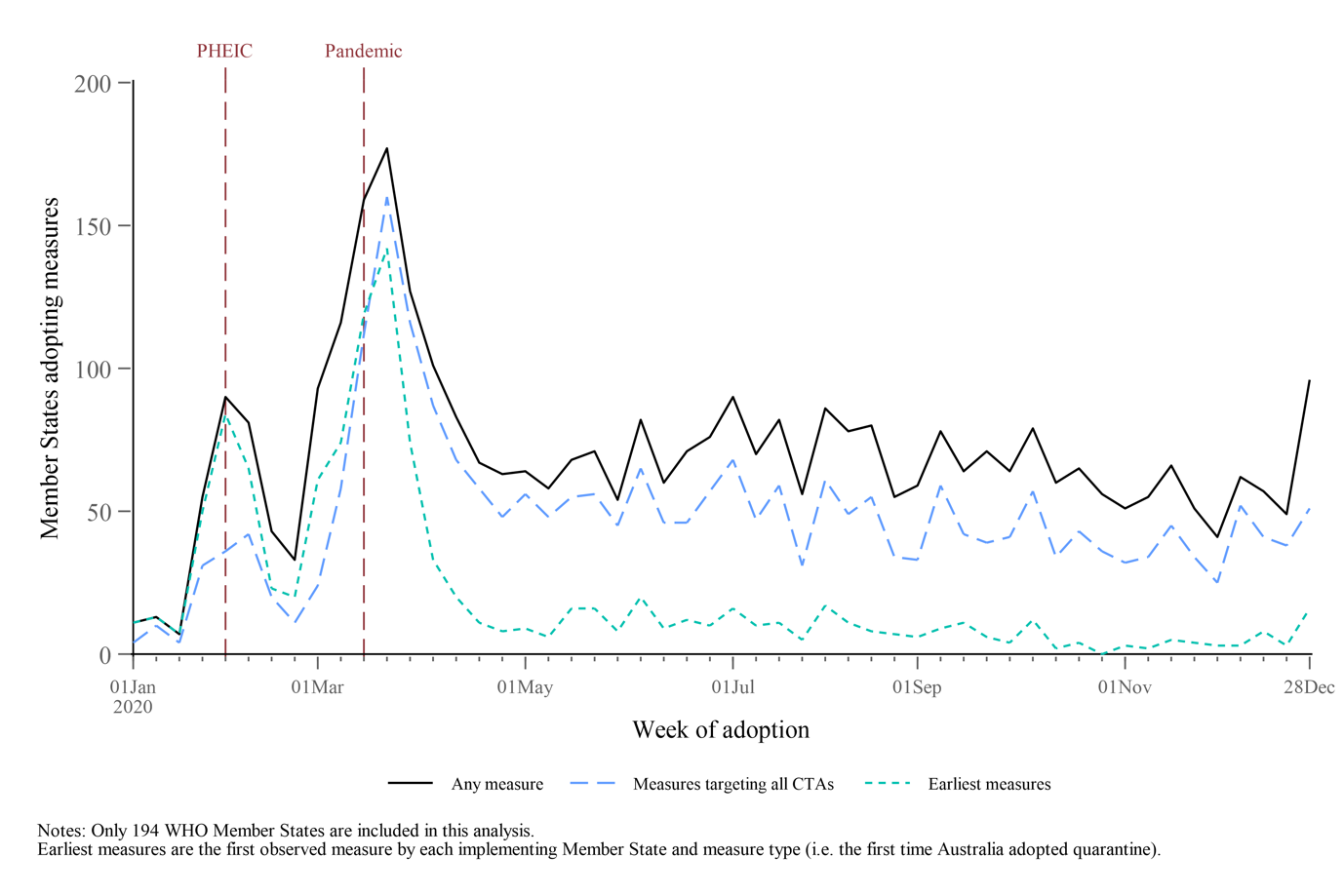


Figure 3: Cumulative proportion of member states adopting specific measure types, by week of adoption and income-level


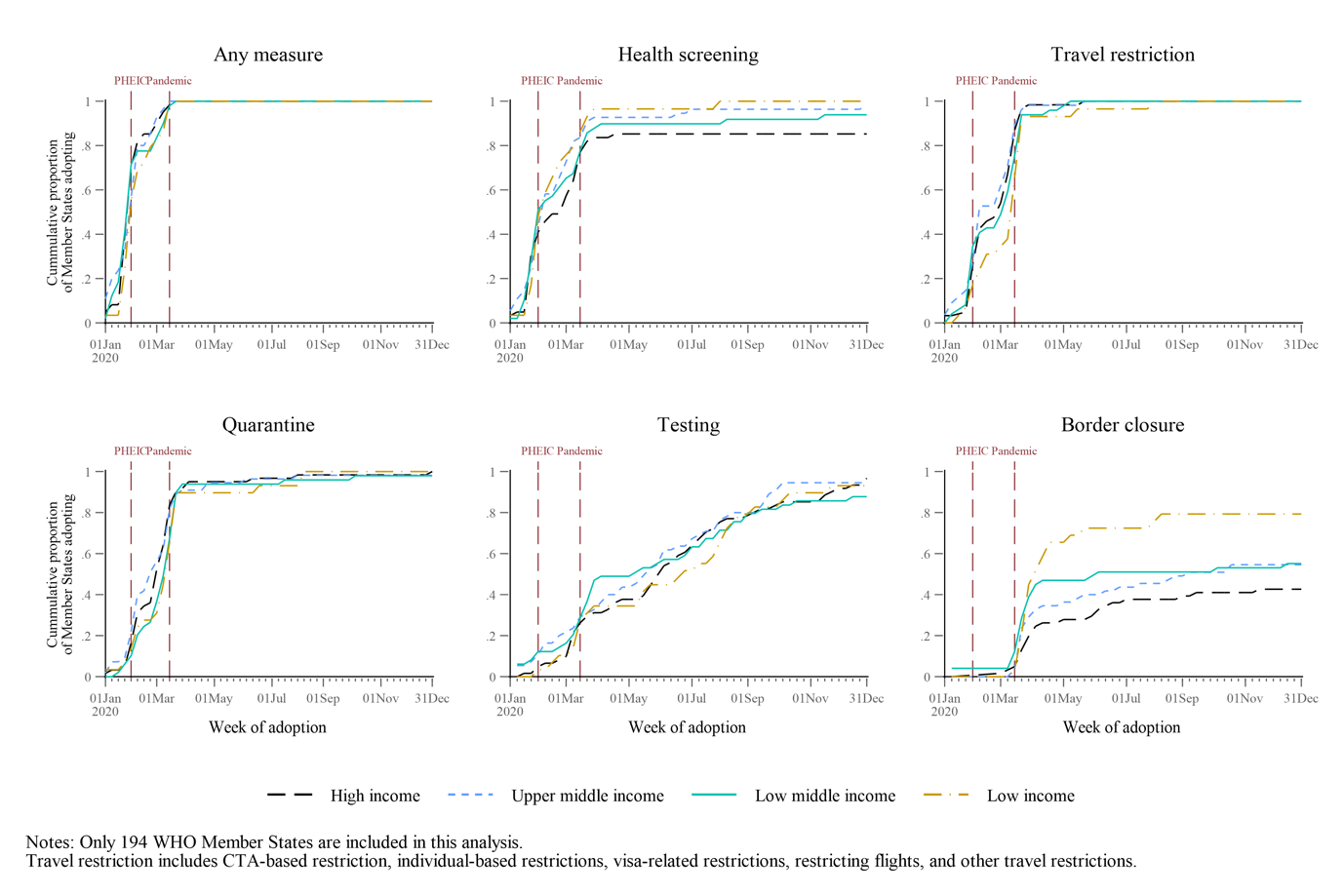


## 6. References

Azevedo, J.P. (2011) "wbopendata: Stata module to access World Bank databases," Statistical Software Components S457234, Boston College Department of Economics. Retrieved February 12, 2023 from <http://ideas.repec.org/c/boc/bocode/s457234.html>

Cheng, C., Desvars-Larrive, A., Ebbinghaus, B., Hale, T., Howes, A., Lehner, L., Messerschmidt, L., Nika, A., Penson, S., Petherick, A., Xu, H., Zapf, A.J., Zhang, Y., Zweig, S.A. (2022) “Capturing the COVID-19 Crisis through Public Health and Social Measures Data Science.” Sci Data 9, 520. <https://doi.org/10.1038/s41597-022-01616-8>

International Organization for Migration (IOM), Jan 16 2023. DTM DTM (COVID-19) Global Mobility Restrictions Overview: March 2020 - January 2023. IOM, Global. Accessed August 1, 2024 from: https://dtm.iom.int/reports/dtm-covid-19-global-mobility-restrictions-overview-march-2020-january-2023

Pareto Software, LLC. (n.d.). World Cities Database | Simplemaps.com. SimpleMaps.Com. Retrieved January 28, 2023, from <https://simplemaps.com/data/world-cities>

1. [↑](#footnote-ref-1)
2. https://www.acaps.org/covid-19-government-measures-dataset [↑](#footnote-ref-2)
3. https://github.com/HopkinsIDD/hit-covid [↑](#footnote-ref-3)
4. https://www.bsg.ox.ac.uk/research/research-projects/covid-19-government-response-tracker [↑](#footnote-ref-4)
5. https://apps.who.int/iris/bitstream/handle/10665/337686/WHO-EURO-2020-1610-41361-56329-eng.pdf?sequence=2&isAllowed=y [↑](#footnote-ref-5)
6. https://www.nature.com/articles/s41562-021-01079-8#Sec6 [↑](#footnote-ref-6)
7. [↑](#footnote-ref-7)
8. Note: In June 2021, the coding team started to use Wayback Machine (https://archive.org/web/) to handle expired and live links. This tool captured a website’s content and saved all updates during the target time periods. Using its API, coders could identify which dates and time (in seconds) Wayback had made a capture of this website. This further allowed coders to locate the measures introduced on a specific date. They could also replace the pre-existing link with an updated link where the accurate information could be accessed. [↑](#footnote-ref-8)
9. Note: Please be reminded that we did not prioritize capturing all international travel measures introduced by the CTAs through a systematic search. However, on occasion coders came across a government website or other high-quality sources where international travel measures of a CTA were tracked and published. In this case, new entries could be inputted by coders. [↑](#footnote-ref-9)
10. Note: The initial coder may use similar expressions to indicate the same issue. [↑](#footnote-ref-10)
